# Eigencentrality based on dissimilarity measures reveals central nodes in complex networks

**DOI:** 10.1038/srep17095

**Published:** 2015-11-25

**Authors:** A. J. Alvarez-Socorro, G. C. Herrera-Almarza, L. A. González-Díaz

**Affiliations:** 1 Departamento de Física, FCFM, Universidad de Chile, Santiago, Chile; 2 Laboratorio de Dinámica No-Lineal y Sistemas Complejos, Centro de Física, Instituto Venezolano de Investigaciones Cientícas, Caracas 1020-A, Venezuela

## Abstract

One of the most important problems in complex network’s theory is the location of the entities that are essential or have a main role within the network. For this purpose, the use of dissimilarity measures (specific to theory of classification and data mining) to enrich the centrality measures in complex networks is proposed. The centrality method used is the eigencentrality which is based on the heuristic that the centrality of a node depends on how central are the nodes in the immediate neighbourhood (like rich get richer phenomenon). This can be described by an eigenvalues problem, however the information of the neighbourhood and the connections between neighbours is not taken in account, neglecting their relevance when is one evaluates the centrality/importance/influence of a node. The contribution calculated by the dissimilarity measure is parameter independent, making the proposed method is also parameter independent. Finally, we perform a comparative study of our method versus other methods reported in the literature, obtaining more accurate and less expensive computational results in most cases.

A large number of systems from nature and others man-made can be described in terms of networks, composed by entities (or nodes) that interact through connections (or links). The topological and statistical properties of the nodes in a network (at microscopic level) tend to be highly heterogeneous, as can be seen by studying their degree distribution, clustering distribution and their degree-degree correlations^1^,^2^,^3^. On the other hand, there are also heterogeneities at mesoscopic and macroscopic levels, e.g., not all the networks have the same hierarchical structure, community structure or topology^4^,^5^,^6^,^7^,^8^. These heterogeneities (at microscopic, mesoscopic and macroscopic levels) have repercussions in the importance of nodes and links in the network. For example, is well known that, in highly modular networks, nodes and links that connect modules or communities, i.e., nodes with neighboring nodes in different communities are more relevant (in terms of global communications) than nodes with neighborhoods fully included in the same community, and links with both extreme nodes in the same module are less relevant than links with extreme nodes in different communities, a fact that has been widely used precisely in the detection of community structures^9^. Thus, there exist nodes that are more important as a result of their position relative to other nodes of the network, giving us relevant information about the properties of the networks. This kind of nodes and links that have a special role in a network are called central with respect to a given role. Thus, one of the ways to address the problem of centrality define first (at least heuristically) the context in which we are talking about “centrality” and then build measures to quantify the definition of centrality used, as in the case of betweenness and closeness^10^. However, although there is no consensus on the concept of centrality (because as we mentioned it depends on the system under study and the context or heuristics behind the “centrality” to be measured), we can propose a definition of centrality that involves all existing definitions.

**Definition.** Let 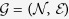 be a network and let 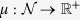 be a measure quantifying a desired property. We say that a node 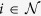 has a *μ*-centrality *k* if *μ*(*i*) = *k*. So that we can talk about closeness - centrality or betweenness - centrality. The reader can find the formula for betweenness and closeness measures in the materials and methods section, equations (11) and (12).

Among the applications of measures of centrality in complex networks we have: (i) in social networks, hubs are related to the most influential people on the network^11^,^12^,^13^, which is of interest to understand the individual and collective social processes and the information spreading in such networks^13^,^14^, (ii) in the protein-protein interaction networks of an organism, central nodes are related to the essential genes, i.e., those genes of an organism that are critical to their survival, which is of broad interest in the research and design of drugs to combat parasitic diseases^15^,^16^,^17^,^18^, (iii) in air or urban traffic networks, the central nodes are associated with optimal points for the spread of diseases, which are of great interest to effectively prevent and control the spread of diseases, putting at these points, checkpoints and vaccination campaigns^19^,^20^,^21^, among many other applications^22^,^23^,^24^. The centrality measures are an attempt to locate these nodes, and their goal is to assign a measure (or rank) to each node so that they can be sorted from highest to lowest centrality. Some of the heuristics and statistics used to define centrality are based on^10^:

1. How connected a node is,
2. How influential a node is in terms of its neighbourhood,
3. How easily a node can propagate an information,
4. How intermediary is a node as a connector between nodes in the network.

In this paper, we propose a new method for finding the centrality of a node in a given network, based on both the sum of the centralities of the nodes in its neighbourhood and on their dissimilarities. The neighbourhood of the node *i* will contribute more to its centrality in the measure in which the nodes of the neighbourhood are more dissimilar. In this work, we will consider that two nodes are dissimilar if they do not share neighbours between them (see Fig. 1).

Let *R* be a network and *A* its adjacency matrix, i.e., *A*_*ij*_ = 1 if the link {*i*, *j*} is in the network and *A*_*ij*_ = 0 otherwise. This matrix indexes 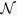.

One method known to find the centrality of the *i*-th node in a network^25^ is based on the following heuristic: “The centrality/relevance/influence of a node is proportional to the sum of centralities of its neighbours”. Mathematically, this can be written as


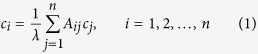


where *c*_*i*_ denotes the centrality of the *i*-th node and 1/*λ* is a constant of proportionality. This leads to the eigenvectors and eigenvalues problem:


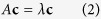


Assuming that *λ* = *λ*_max_ = *ρ*(*A*) is the spectral radius of the adjacency matrix of the network, i.e., the largest eigenvalue, then by the Perron-Frobenius theorem^26^ there exists a unique nonnegative eigenvector **c** that satisfies the above equation, obtaining the well-known measure of eigencentrality^25^. Note that to know how central or influential the *i*-th node is in the network, we only need to know the value of the *i*-th entry of the vector **c**.

The advantages of this approach are: (i) uses local information because the centrality of a node depends explicitly on the centrality of its neighbours, (ii) uses the global information of the network through successive couplings (i.e., the centralities of the nodes in the neighbourhood of a node also depends on their neighbours, and so on), involving all network nodes in the centrality of a given node, (iii) one can analyse large networks quickly, since there are a variety of numerical methods for calculating eigenvalues and eigenvectors fairly efficiently^27^.

The main disadvantage of this approach lies precisely in its heuristics, since it assumes that all nodes in the neighbourhood of *i*-th node contribute equally to its centrality, which is in general false (see Fig. 1), leading to a poor ranking, as will be discussed in the results section. Therefore, it is necessary to reformulate this heuristic.

## Contribution Centrality

**Figure 1:**
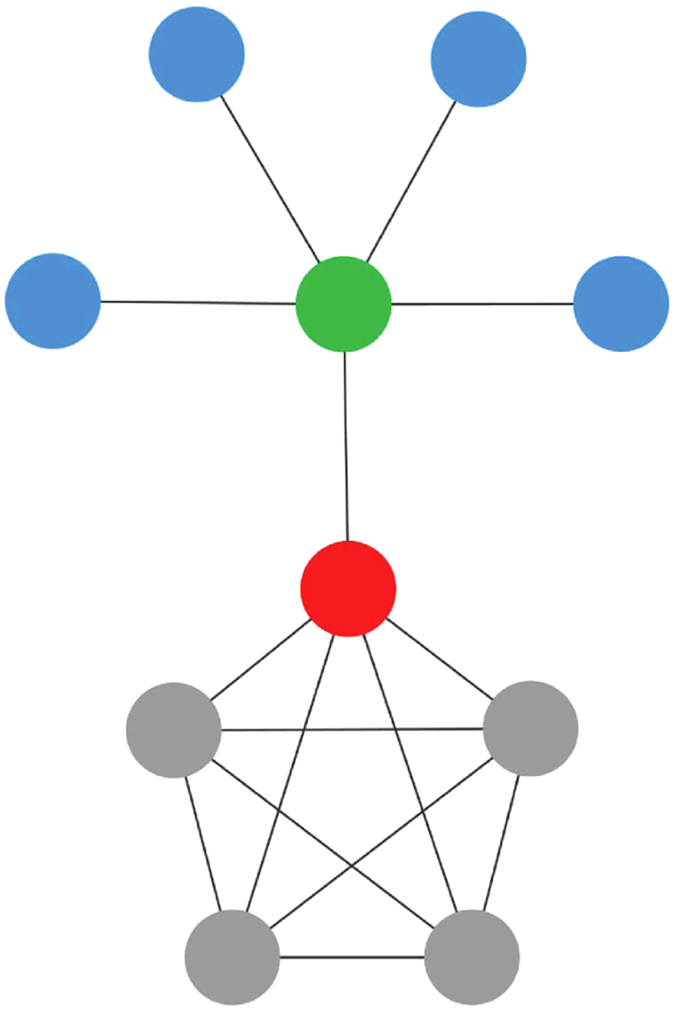
In the illustrated network, green and red node are dissimilar because they do not share neighbors between them. The red node reaches the blue nodes only through the green node and therefore its contribution to the centrality of red node is greater.

To illustrate the essence of our method, consider the network of Fig. 1. For the red node, the information, relevance or centrality that brings a particular gray node is poor, because the red node can access the rest of the gray nodes directly, without any intermediary (this is because they have almost the same neighbours). However, the green node is essential for red node, because without it, the red node could not access to the blue nodes. One way to quantify this is through a structural dissimilarity measure, e.g, Jaccard dissimilarity^28^, given by


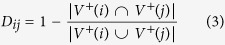


that allows us to measure the difference between the neighbourhoods of two nodes *i*, *j* given. Other measures of structural and dynamical dissimilarities are discussed in the supplementary material. Thus, we can weigh the contribution made by each node *j* in the neighbourhood of a node *i* by


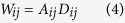


This allows us to propose the following heuristic: “the centrality of a node is proportional to the sum of the centralities of the nodes in its neighbourhood, weighed by their contributions.” Mathematically, that is


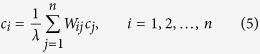


leading us to the eigenvalues - eigenvectors problem


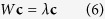


where 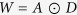, and 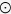 is the coordinate to coordinate product of matrices *A* and *D*. Note that *A* and *D* are non-negative matrices, so we can use the Perron-Frobenius theorem to ensure that the above problem has a unique solution for *λ* = *λ*_*max*_ with **c** non-negative, allowing us to infer the centrality of each node in the network. Thus,


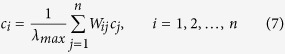


## Results

Natural networks that have information about the centrality of the nodes are commonly used as benchmarks^29^, because, to date, it has not reported a quantitative methodology to study the accuracy of centrality measures used in the detection of the most important nodes, since the concept of importance depends strongly on the system under study and the topological properties of the network considered. However, in order to have information on the runtime of our algorithm, a group of scale-free networks, Barabási - Albert type, with parameter *k* = 2, was studied, taking 21 logarithmically spaced values of *N*, 10 networks and then averaging runtime^5^. Analysed networks from Barabási-Albert model have a number of nodes from 10^2^ to 10^5^ nodes. The computation time spent for the analysis of a network of 10^5^ nodes proved to be a few minutes, as can be seen in the supplementary material.

Motivated by the fact that centrality measures have been used interchangeably to locate the most important nodes in a network^12^,^22^,^23^, although they measure different properties, we performed a comparative study between our measure and some of the most common centrality measures in different networks.

### Florentine Marriages Network

This network was taken from^30^, it was constructed through data from historical documents on the social relations among renaissance Florentine families. In^31^ is provided evidence that support why the Medici were the most powerful family in the early fifteenth century in Florence.

**Figure 2:**
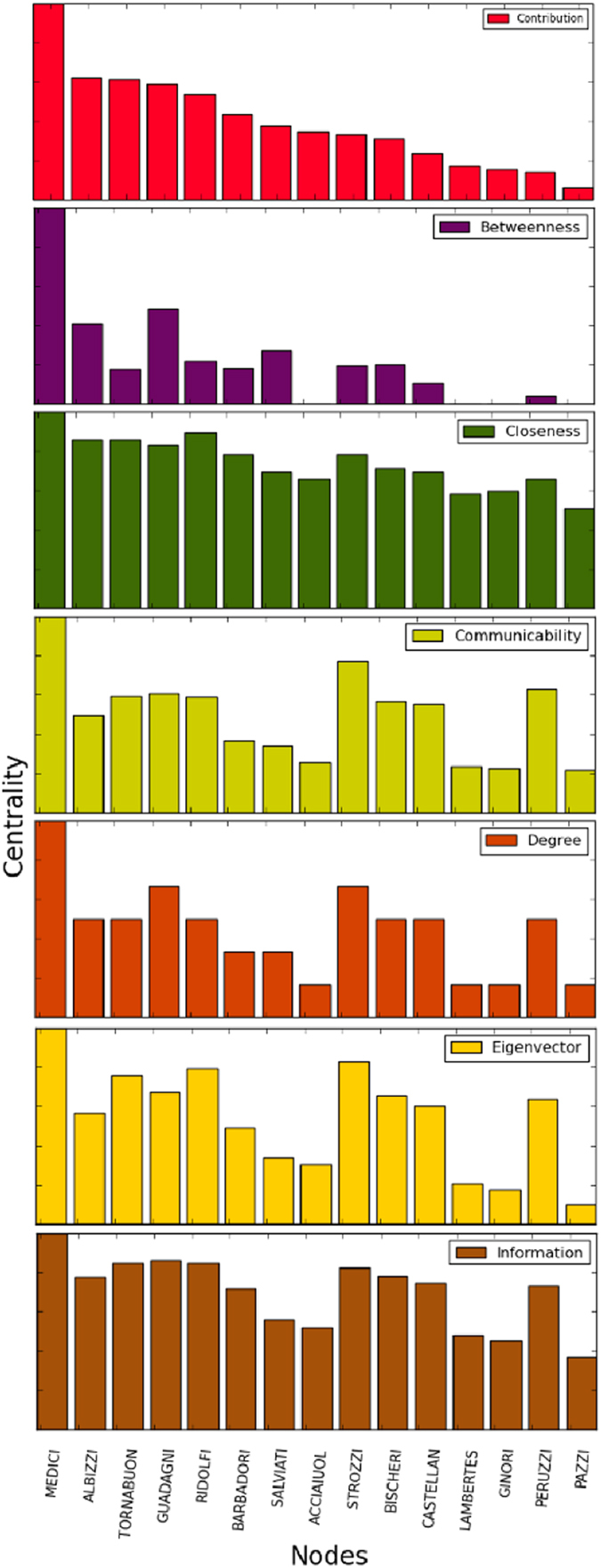
Distributions corresponding to the diverse measures of centrality of the Florentine marriages network.

**Figure 3:**
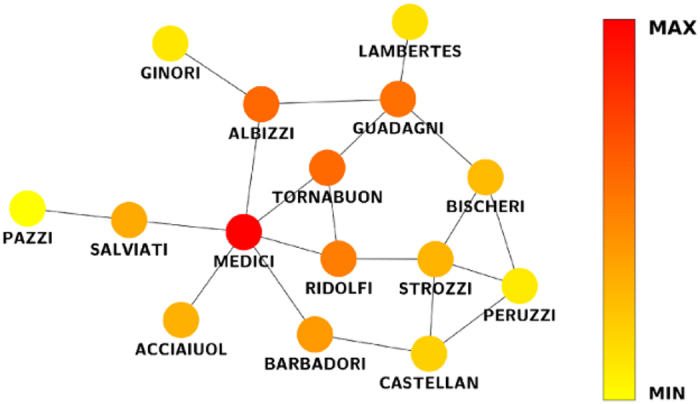
Florentine marriages network.

Applying (7) to this network, we find that the most central node is associated with the Medici family. In Fig. 2, we note that there is a difference between the ranking produced by (7) and the other centrality measures, mainly emphasizing the difference between the results obtained with our method and those obtained with eigencentrality, although both measures have similar heuristics. In Fig. 3, the network is illustrated with the different centrality values produced by (7).

### Zachary’s Karate Club Network

This network was taken from^32^ where the nodes are members of a university karate club, and the links represents the presence of ties among the members of the club. For our proposes, we take only the topology, without the weight of the links.

**Figure 4:**
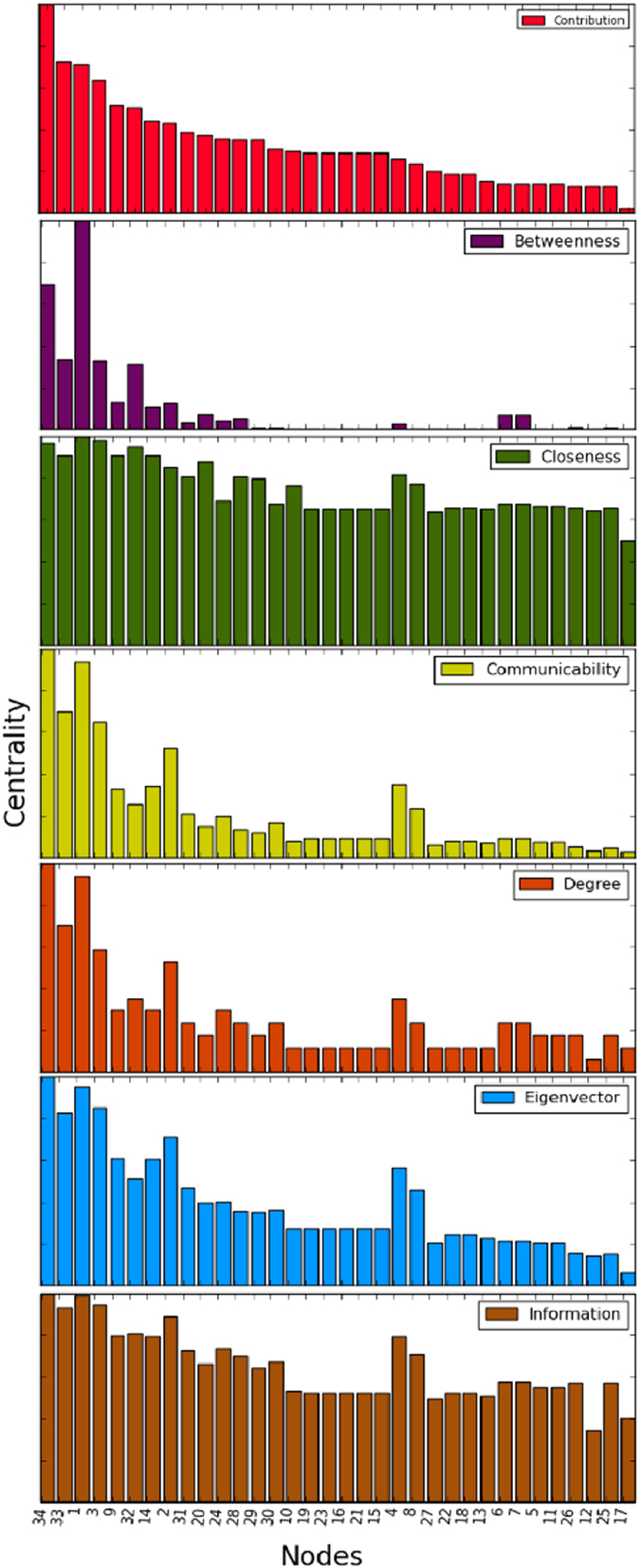
Distributions corresponding to the diverse centrality measures of the network of Zachary’s karate club.

**Figure 5:**
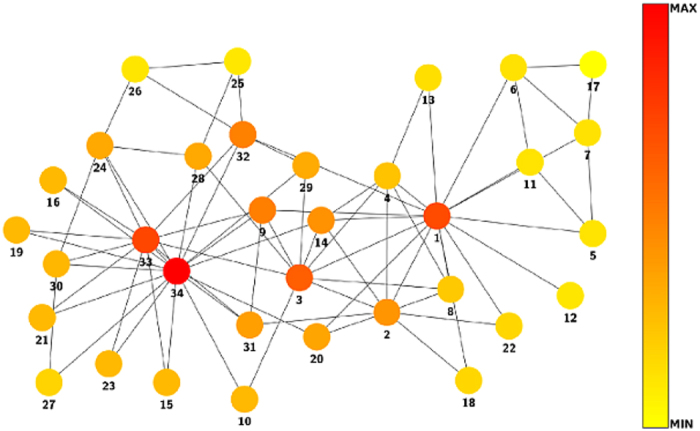
Zachary’s karate club network.

It is known that the most important nodes in this network are 34, 33 and 1, being 34 the president of the students’ club, 33 is the vice-president, and 1 the karate sensei, hired by the club. Applying our method to this network, we obtained the same result. In this sense, the contribution centrality provides a more suitable ranking to the importance of the nodes than closeness and betweenness because in this particular network the importance of a node is not directly related with this concepts. Note also that 33 as vice president, inherits the role of 34 in his absence, so his importance in decision-making in the club and his direct connection to 34 makes him the second most important node according to our measure. In Fig. 4, we can see a clear difference between the ranking produced by the contribution centrality and other methods compared here. In Fig. 5, the network is illustrated with the different centrality values produced by (7).

### Les Miserables Coappearances Network

Finally, we take the coappearances network of characters in Victor Hugo’s novel “Les Miserables” taken from^33^. The nodes represent characters and links that connect any pair of characters that appear in the same chapter of the book. We are staying only with the topology, ignoring the number of such coappearances.

**Figure 6:**
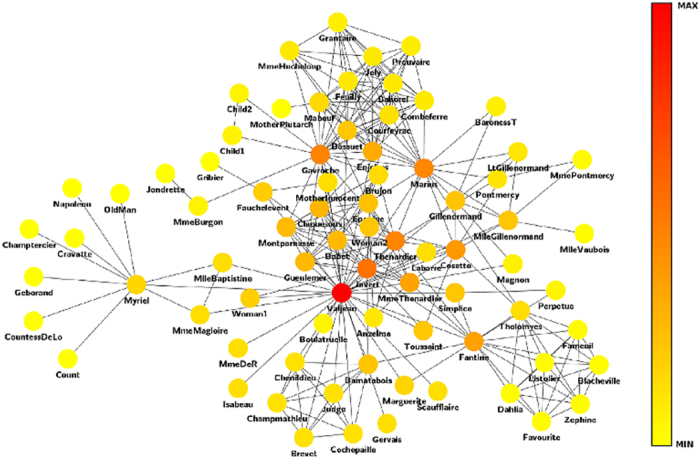
Les Miserables coappearances network.

In this network, the contribution centrality achieves again, a more suitable ranking than other centrality measures considered. Table 1 shows the first 10 nodes sorted by relevance according to different centrality measures. We found that communicability and eigenvector centrality fail to detect Valjean as the most important node. For the centralities betweenness, closeness and degree, Valjean is detected as the main node, but they are not able to find the second most important node, which is undoubtedly Javert, co-protagonist and antagonist character Valjean in the novel. The information centrality produced good results in this case, however, has misclassified to Enjolras when placed over Marius and Cosette, which does not happen with the contribution centrality. Regarding the latter, it is also noted that Cosette does not appear in the top 10 major nodes in some centralities, for example, eigenvector, degree, communicability, betweenness. Thus, the contribution centrality provides a much better ranking of the characters in the novel Les Miserables, than other methods, just taking the topological structure of the network, not the frequency of co-occurrences. In Fig. 6, the network is illustrated with the different centrality values produced by (7).

## Discussion

**Figure 7:**
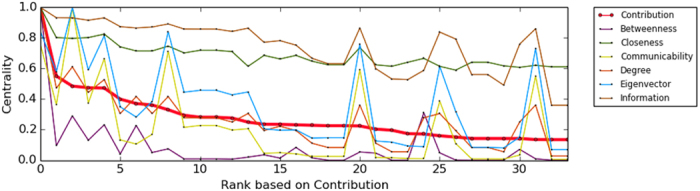
Distributions corresponding to the diverse measures of centrality of the Les Miserables coappearances network.

In this paper, a general methodology that uses structural dissimilarity measures to enrich centrality methods in complex networks is proposed, illustrating this through eigencentrality method and Jaccard dissimilarity. The combination exploits local and global information network, contributing more to the centrality the most dissimilar neighbours of given node. Note the difference between the eigencentrality method and our method (see Table 1), indicating the important effect of the inclusion of dissimilarity measures as weight parameters when considering the contribution of the neighbors of a given node to its centrality. The eigencentrality method could not detect Valjean as the most important character in the novel Les Miserables, which our method achieved satisfactorily. Overall our measure of centrality behaved differently than the rest of the measures of centrality studied here, which can be clearly seen in Figs 2, 4 and 7. Although compared centrality measures measure different properties, all have been used for detection of the key nodes in the network under consideration^12^,^22^,^23^. In this sense, we compare the results obtained with different centrality measures in each network. Note that our measure tends to adapt well to local and global topological properties of networks studied in this work.

Our method can be extended directly to weighted and directed networks, considering, e.g, the Tanimoto coefficient^34^, since the only restriction imposed by our methodology is that the *W* matrix is non-negative to ensure existence and uniqueness of the non-negative eigenvector of centrality. The method is quite simple and powerful. Analysis of networks of over a million nodes was achieved quickly, as can be seen in the supplementary material. Note that the coupling of the matrices *A* and *D* for the construction of *W* ends in some cases in a less dense matrix than *A*, since if two nodes have exactly the same neighbourhood, the contribution that one provides the other will be zero, eliminating the associated entry in the *W* matrix, accelerating the calculation of eigenvectors and reducing memory cost. The contribution centrality method can be applied to the development of recommender systems and search engines because metadata nodes (e.g, information from a web page, keywords, number of clicks, etc.) can be introduced into the dissimilarity measure, to obtain a ranking of the nodes is terms of its relevance, similar to Google PageRank^35^. Note that the strategy of introducing metadata nodes within dissimilarity measure is very general and does not depend on the type of system under study. Finally, the contribution centrality method was applied to social, biological, transport and artificial networks, obtaining in all cases excellent results, as shown in the supplementary material.

## Materials and Methods

In the following, 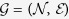 will denote a unweighted, undirected and connected network, with 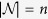 nodes and 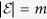 links and *A* will denote the adjacency matrix of the network 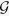.

### Degree Centrality

The degree centrality^36^ is the most simple centrality measure of all. Its heuristic is based on the idea that the most connected node is the most central. Hence, we can consider the centrality of a node *i* as proportional to the degree (or number of connections) of that node, i.e.,


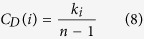


In terms of the adjacency matrix, we can write the above equation as


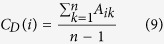


### Eigenvector Centrality

The heuristic behind the eigencentrality measure^25^ is based on the key idea that the centrality of a node is proportional to the sum of centralities of its neighbourhood and therefore, a node connected to central nodes, will also be central. As explained in the introductory section of this paper, this heuristic leads us to calculate the centrality of a node *i* by an equation of the form


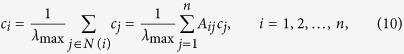


where *λ*_max_ is the largest eigenvalue of the adjacency matrix *A*. Note that the above equation leads directly to the eigenvector problem:


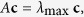


where **c** exist and it is positive as consequence of the Perron-Frobenius theorem^26^.

### Betweenness Centrality

This is one of the most popular measures of centrality in the literature^37^ and is based on the idea that nodes for which more information flows, will be higher values of centrality, under the assumption that the information always travels along the shortest paths connecting any two nodes in the network. Mathematically, this is


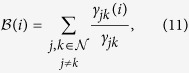


where *γ*_*jk*_ is the number of shorter paths ranging from the node *j* to node *k* and *γ*_*jk*_(*i*) is the number of shorter paths ranging from the node *j* to node *k* and passing through the node *i*.

### Closeness Centrality

The heuristic principle behind this measure is based on the concept of closeness^38^. A node is central when it can reach any node in the network in few steps, i.e., is closer to all nodes. Thus, the node with higher centrality value will be one with the lowest average length of shortest paths, i.e.,


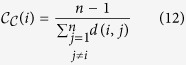


where *d*(*i*, *j*) is the shortest path distance between the nodes *i* and *j*. In the case where the network is unconnected, closeness centrality is calculated separately for each connected component is calculated separately.

### Information Centrality

The information centrality^39^ is based on the study of how information flows between all pairs of nodes in the network. For a given node *i*, the information centrality is calculated through the harmonic mean of the combined paths information, for all nodes *j* in the network, meaning by combined path between *i* and *j* the set of all paths joining this pair of nodes. Thus, we have mathematically


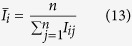


The information of a path is defined as the inverse of its length. If paths that make up a combined path are independent (i.e not shared links) then the combined path information *I*_*ij*_ is given by the sum of the information of the paths that compose it. Otherwise, it is necessary to calculate the matrix *D*(*i*, *j*) containing the number of links that share the paths in combined path and its information is given by 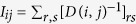.

### Communicability Centrality

The communicability centrality^40^,^41^, also called subgraph centrality exploit all closed paths of all lengths that start and end at a node *i*, having greater influence on the centrality of node *i* paths with shorter length. Mathematically, the communicability of a node 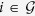 is calculated by the exponential of the adjacency matrix of the network as:


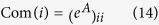


## Additional Information

**How to cite this article**: Alvarez-Socorro, A. J. *et al.* Eigencentrality based on dissimilarity measures reveals central nodes in complex networks. *Sci. Rep.* **5**, 17095; doi: 10.1038/srep17095 (2015).

## Supplementary Material

Supplementary Information

## Figures and Tables

**Table 1:** First 10 nodes sorted by relevance according to different measures of centrality in the network of co-occurrences of characters in the novel by Victor Hugo’s Les Miserables.

Index	Contribution	Betweenness	Closeness	Communicability	Degree	Eienvector	Information
1	Valjean	Valjean	Valjean	Gavroche	Valjean	Gavroche	Valjean
2	Javert	Myriel	Marius	Valjean	Gavroche	Valjean	Javert
3	Gavroche	Gavroche	Thenardier	Enjolras	Marius	Enjolras	Marius
4	Thenardier	Marius	Javert	Marius	Javert	Marius	Gavroche
5	Marius	Fantine	Gavroche	Bossuet	Thenardier	Bossuet	Thenardier
6	Cosette	Thenardier	Enjolras	Courfeyrac	Fantine	Courfeyrac	Enjolras
7	Fantine	Javert	Cosette	Bahorel	Enjolras	Bahorel	Cosette
8	MmeThenardier	MlleGillenormand	Bossuet	Joly	Courfeyrac	Joly	MmeThenardier
9	Enjolras	Enjolras	Gueulemer	Combeferre	Bossuet	Feuilly	Bossuet
10	Claquesous	Tholomyes	Babet	Feuilly	Joly	Combeferre	Fantine
