# Supplementary material for: Eigencentrality based on dissimilarity measures reveals central nodes in complex networks
Source: Sci Rep. 2015 Nov 25;5:17095. doi: 10.1038/srep17095 (PMC4658528; doi:10.1038/srep17095)
Supplement: Supplementary Information [file srep17095-s1.pdf]

Eigencentrality based on dissimilarity measures reveals central nodes in complex networks

Alvarez-Socorro A. J.[1,2], Herrera-Almarza G. C.[1,2], González-Díaz, L. A.[2]

[1]Departamento de Física, FCFM, Universidad de Chile, Santiago, Chile.

[2]Laboratorio de Dinámica No-Lineal y Sistemas Complejos, Centro de Física, Instituto Venezolano de Investigaciones Científicas, Caracas 1020-A, Venezuela.

Correspondence and requests for materials should be addressed to A.J.A-S (alvarez.ciencia@gmail.com)

Supplementary Information

Contents

1 Structural Dissimilarity Measures **2**
1.1 Jaccard Dissimilarity 2
1.2 Meet/Min Dissimilarity or Topological Overlapping 2
1.3 Geometric Dissimilarity 3
1.4 Sørensen-Dice Dissimilarity 3
1.5 Maryland Bridge Dissimilarity 3
1.6 Czekanovski-Dice Dissimilarity 3
1.7 Korbel Dissimilarity 4
1.8 Others possible measures 4
1.8.1 A Dynamical Dissimilarity 4
1.8.2 A Random Walk Dissimilarity 5

2 Comparative analysis of the structural dissimilarity measures **5**

3 Datasets **7**
3.1 Florentine Marriages Network 7
3.2 Zachary's Karate Club Network 10
3.3 Les Miserables Coappearances Network 13
3.4 Dolphin social network 16
3.5 Terrorist Network 19
3.6 Airport USA 97 23

4 Runtime performance **26**

1 Structural Dissimilarity Measures

Given two nodes i and j, we are interested in studying how dissimilar are among them, by comparing their respective neighbourhoods. Thus, two nodes with the same neighbourhood would have dissimilarity equal to zero, whereas if two nodes have a very different neighbourhoods, the associated dissimilarity value will be greater than zero (in this work we will use normalized dissimilarities so that its maximum value is 1). We will compare the level of dissimilarity between two nodes through their neighbourhoods, and the measures that allow us to make this comparison will be called structural dissimilarities.

Note that given a node i, we can weigh the local importance of each node in the neighbourhood of i through their its dissimilarity level. The greater the dissimilarity between node i and a node j the greater the number new contacts that i will reach through j, so j allows i to spreading information, virus, opinions, roumors, signals, etc., beyond that of its immediate neighborhood, i.e., j "opens the world" to i.

If we have a normalized similarity measure $S(\cdot,\cdot)$, we can build a normalized dissimilarity measure $D(\cdot,\cdot)$ taking

$$D(\cdot,\cdot) = 1 - S(\cdot,\cdot) \tag{1}$$

In the following, we will test a set of structural dissimilarities popular in literature.

1.1 Jaccard Dissimilarity

Jaccard similarity [8] is defined as the proportion of shared nodes between i and j nodes on the number of nodes in both neighbourhoods. Inclusive neighbourhood $V^+(i) = V(i) \cup \{i\}$ instead of using the simple neighbourhood is necessary because in the case of two nodes connected between them and not connected to any other node, the dissimilarity that considers inclusive neighbourhoods gives us that such nodes are completely similar, while the simple neighbourhood fails to capture the direct connection between nodes and produce a dissimilarity equals to 1. Mathematically, Jaccard dissimilarity is defined by equation (1) as [7]:

$$D_{ij} = 1 - \frac{|V^+(i) \cap V^+(j)|}{|V^+(i) \cup V^+(j)|} \tag{2}$$

1.2 Meet/Min Dissimilarity or Topological Overlapping

Topological Overlapping [8] or *Meet/Min similarity* [9] is defined as the ratio of shared nodes between neighbourhoods of the nodes i and j, and the minimum number of nodes in some of the neighbourhoods. Mathematically we build the associated dissimilarity is

$$D_{ij} = 1 - \frac{|V^+(i) \cap V^+(j)|}{\min\{|V^+(i)|, |V^+(j)|\}} \tag{3}$$

Note that although this measure is mathematically like Jaccard dissimilarity, its behaviour is generally quite different. Note that if the inclusive neighbourhood of a node is entirely contained in the inclusive neighbourhood of another, then two nodes have zero dissimilarity, which is not true in the case of the Jaccard's dissimilarity. This shows the importance of the normalization factor when calculating the dissimilarity between two nodes in a network.

1.3 Geometric Dissimilarity

Given the nodes i and j in the network, *the geometric similarity* [8] between these nodes corresponds to the ratio of the square of the number of nodes that share the neighbourhoods of nodes i and j and the product of the number of nodes in each of the neighbourhoods. Mathematically, the dissimilarity will be

$$D_{ij} = 1 - \frac{|V^+(i) \cap V^+(j)|^2}{|V^+(i)| \cdot |V^+(j)|} \tag{4}$$

Note that both the numerator and denominator are different from the above two measures.

1.4 Sørensen-Dice Dissimilarity

Given two nodes i and j, the *Sørensen-Dice dissimilarity* (SD) [10] between these nodes is given by

$$D_{ij} = 1 - \frac{2|V^+(i) \cap V^+(j)|}{|V^+(i)| + |V^+(j)|} \tag{5}$$

with range between 0 and 1. Again, this measure is like Jaccard, nevertheless behaves different, in fact, one can show that this dissimilarity is not a metric, since does not satisfy the triangle inequality, unlike Jaccard dissimilarity.

1.5 Maryland Bridge Dissimilarity

The *Maryland Bridge dissimilarity* (MB) [11] has been used mainly in the genome classification problem and is defined as

$$D_{ij} = 1 - \frac{1}{2}\left(\frac{|V^+(i) \cap V^+(j)|}{|V^+(i)|} + \frac{|V^+(i) \cap V^+(j)|}{|V^+(j)|}\right) \tag{6}$$

Note that the similarity associated with (6) measures the average proportion of the overlap between the respective neighbourhoods.

1.6 Czekanovski-Dice Dissimilarity

The *Czekanovski-Dice dissimilarity* (CD) [12] is given by

$$D_{ij} = \frac{|V^+(i) \triangle V^+(j)|}{|V^+(i) \cup V^+(j)| + |V^+(i) \cap V^+(j)|} \tag{7}$$

where

$$V^+(i) \triangle V^+(j) := \big(V^+(i) \setminus V^+(j)\big) \cup \big(V^+(j) \setminus V^+(i)\big) \tag{8}$$

is the symmetric difference between inclusive neighbourhoods. It has been mainly has been used in protein-protein interaction data mining.

1.7 Korbel Dissimilarity

Given two nodes i and j we define the *Korbel dissimilarity* [13] as

$$D_{ij} = 1 - \frac{\sqrt{|V^+(i)|^2 + |V^+(j)|^2}}{\sqrt{2}|V^+(i)||V^+(j)|} \tag{9}$$

This measure has been used by Korbel in order to take advantage of the information from complete genomes and classify species in phylogeny.

1.8 Others possible measures

There are other dissimilarity measure that are not explicitly based in the neighbourhoods of nodes to compare. For example, we can compare (i) the dynamical behaviour of a node in a system of oscillators coupled (in general coupled maps) on the network under study or (ii) consider the dissimilarity of the paths travelled of random walks of k steps that starting from nodes i and j, respectively. In the following, we will discuss these dissimilarity measures.

1.8.1 A Dynamical Dissimilarity

Consider the system of coupled maps on the network:

$$x_i^{t+1} = (1-\epsilon)f(x_i^t) + \frac{\epsilon}{|V(i)|}\sum_{j=1}^{n} A_{ij} f(x_j^t) \tag{10}$$

where A is the adjacency matrix and ϵ is the coupling strength. Given a random initial condition (initial state vector), if the system is allowed to evolve a time series for each node will be obtained. We can measure how similar are this time series (and therefore the associated nodes), using e.g., Pearson, Spearman or Kendall correlation coefficients [14] or normalized mutual information [15]. Thus, we propose quantify the level of relationship between the dynamics of both nodes particularly using Pearson correlation coefficient, given by

$$\begin{aligned}
\rho_{ij} &= \rho(\mathcal{W}^i(\mathbf{x_0}, t_1, t_2), \mathcal{W}^j(\mathbf{x_0}, t_1, t_2)) \\
&= \frac{\sum_{t=t_1}^{t_2} x_i^t x_j^t - n\bar{x}_i\bar{x}_j}{n\sigma(\mathcal{W}^i(\mathbf{x_0}, t_1, t_2))\sigma(\mathcal{W}^j(\mathbf{x_0}, t_1, t_2))} \\
&= \frac{n\sum_{t=t_1}^{t_2} x_i^t x_j^t - \sum_{t=t_1}^{t_2} x_i^t \sum_{t=t_1}^{t_2} x_j^t}{\sqrt{n\sum_{t=t_1}^{t_2}(x_i^t)^2 - (\sum_{t=t_1}^{t_2} x_i^t)^2}\sqrt{n\sum_{t=t_1}^{t_2}(x_j^t)^2 - (\sum_{t=t_1}^{t_2} x_j^t)^2}}
\end{aligned} \tag{11}$$

where $\mathcal{W}^i(\mathbf{x_0}, t_1, t_2)$ represents an observation window in the time series associated to node i, with initial condition $\mathbf{x_0}$ and taking values between the time steps t_1 and t_2, and $\sigma(\mathcal{W}^i(\mathbf{x_0}, t_1, t_2))$ is the standard deviation of $\mathcal{W}^i(\mathbf{x_0}, t_1, t_2)$. The correlation coefficient in (11) ranges in the interval $[-1, 1]$, characterizing the behaviour of a time series based on the behaviour of the other one in the following way:

1. If $\rho_{ij} = 1$, there is a perfect positive correlation. In this case, the index indicates a total dependency between the two time series known as direct relationship: when one increases, so does the other at a constant rate.

2. If $0 < \rho_{ij} < 1$, there is a positive correlation, i.e., the time series have similar but not identical behaviour.

3. If $\rho_{ij} = 0$, there is no linear relationship. But this does not necessarily imply that the variables are independent: nonlinear relationships may exist between the two variables.

4. If $-1 < \rho_{ij} < 0$, there is a negative correlation.

5. If $\rho_{ij} = -1$, there is a perfect negative correlation. The index indicates total dependence between the two variables called inverse relationship: when one increases the other decreases at a constant rate.

To construct a dissimilarity measure between nodes, it is proposed

$$D_{ij} = 1 - \frac{\rho_{ij} + 1}{2} \tag{12}$$

which can measure how dissimilar two nodes are, through the level of correlation between the associated time series. Note that $D_{ij} = 0$ if the time series of the corresponding nodes are perfectly correlated and $D_{ij} = 1$ if there is a perfect negative correlation between time series.

1.8.2 A Random Walk Dissimilarity

Given two nodes in a network, we can try to measure its dissimilarity by comparing its corresponding travelled paths obtained by leaving evolve, as many times as necessary, a uniform random walker that start from each node. Although this strategy has been used in the problem of locating central nodes in networks [26, 27] mainly using the frequency of visits as a criterion of centrality, in this paper we propose the use of measures like Pearson coefficient, mutual information or set-theoretic dissimilarities measures over the trayectories in order to feeding centrality methods in complex networks, particularly the proposed in our work. It is noteworthy that this strategy has also been widely exploited in the context of detection of communities [28, 29].

Dynamical and random walk dissimilarities measures require a deeper study of their behaviour, because their high computational cost and the number of realizations required could make them impractical for big data analysis over networks, nevertheless we propose them as interesting alternatives to structural dissimilarities.

2 Comparative analysis of the structural dissimilarity measures

In order to test the performance of each structural dissimilarity measures presented here, we use as benchmark Les Misserables network. In the Table 1, we show the ranking obtained by our method using each one of structural dissimilarity measures.

Ranking	Jaccard	Meet/Min	SD	MB	CD	Korbel
1	Valjean	Valjean	Valjean	Valjean	Valjean	Valjean
2	Javert	Marius	Javert	Javert	Javert	Marius
3	Gavroche	Thenardier	Marius	Marius	Marius	Javert
4	Thenardier	Javert	Gavroche	Thenardier	Gavroche	Thenardier
5	Marius	Fantine	Thenardier	Gavroche	Thenardier	Gavroche
6	Cosette	Gavroche	Cosette	Cosette	Cosette	Cosette
7	Fantine	Cosette	Fantine	Fantine	Fantine	Fantine
8	MmeThenardier	MmeThenardier	MmeThenardier	MmeThenardier	MmeThenardier	MmeThenardier
9	Enjolras	Enjolras	Enjolras	Enjolras	Enjolras	Enjolras
10	Claquesous	Eponine	Claquesous	Claquesous	Claquesous	Eponine
11	Gueulemer	Tholomyes	Simplice	Eponine	Simplice	Claquesous
12	Babet	Claquesous	Montparnasse	Gueulemer	Montparnasse	Gueulemer
13	Montparnasse	Bossuet	Babet	Babet	Babet	Babet
14	Eponine	Babet	Gueulemer	Montparnasse	Gueulemer	Montparnasse
15	Gillenormand	Gueulemer	Toussaint	Bossuet	Woman2	Bossuet
16	Bamatabois	Bamatabois	Woman2	MlleGillenormand	Toussaint	MlleGillenormand
17	MlleGillenormand	MlleGillenormand	Gillenormand	Gillenormand	Gillenormand	Gillenormand
18	Simplice	Mabeuf	Bamatabois	Bamatabois	Bamatabois	Tholomyes
19	Woman2	Gillenormand	MlleGillenormand	Simplice	MlleGillenormand	Bamatabois
20	Toussaint	Montparnasse	Fauchelevent	Tholomyes	Fauchelevent	Mabeuf
21	Bossuet	Courfeyrac	Woman1	Fauchelevent	Woman1	Simplice
22	Fauchelevent	Myriel	Bossuet	Myriel	Bossuet	Fauchelevent
23	Woman1	Fauchelevent	Myriel	Woman2	Myriel	Courfeyrac
24	Marguerite	Pontmercy	Marguerite	Toussaint	Marguerite	Myriel
25	Myriel	Simplice	Eponine	Mabeuf	Eponine	Brujon
26	Mabeuf	Brujon	MotherInnocent	Woman1	MotherInnocent	Pontmercy
27	Brujon	Magnon	Gervais	Courfeyrac	Scaufflaire	Woman2
28	MmeMagloire	Joly	Scaufflaire	Brujon	Isabeau	Toussaint
29	MlleBaptistine	Bahorel	Labarre	Marguerite	MmeDeR	Joly
30	MotherInnocent	MmeBurgon	Isabeau	Pontmercy	Gervais	Bahorel
31	Tholomyes	Feuilly	MmeDeR	MlleBaptistine	Labarre	Combeferre
32	Courfeyrac	Combeferre	MmeMagloire	MmeMagloire	MmeMagloire	Feuilly
33	Gervais	MmePontmercy	MlleBaptistine	MotherInnocent	MlleBaptistine	Woman1
34	Scaufflaire	Grantaire	Tholomyes	Isabeau	Tholomyes	Marguerite
35	Labarre	MotherPlutarch	Champmathieu	Scaufflaire	Judge	MmeHucheloup
36	Isabeau	Fameuil	Brevet	MmeDeR	Champmathieu	LtGillenormand
37	MmeDeR	Cochepaille	Cochepaille	Gervais	Chenildieu	Anzelma
38	Judge	Zephine	Judge	Labarre	Brevet	Magnon
39	Chenildieu	Brevet	Chenildieu	Bahorel	Cochepaille	MlleBaptistine
40	Cochepaille	Anzelma	Mabeuf	Joly	Mabeuf	MmeMagloire
41	Champmathieu	Labarre	Brujon	Combeferre	Brujon	Prouvaire
42	Brevet	Geborand	Pontmercy	Feuilly	Pontmercy	MmeBurgon
43	Pontmercy	LtGillenormand	Courfeyrac	Anzelma	Courfeyrac	MotherInnocent
44	Anzelma	Count	LtGillenormand	LtGillenormand	LtGillenormand	Grantaire
45	LtGillenormand	Isabeau	Anzelma	Champmathieu	Anzelma	Judge
46	MmeHucheloup	Champtercier	MmeHucheloup	Chenildieu	MmeHucheloup	Champmathieu
47	Feuilly	MlleBaptistine	BaronessT	Judge	BaronessT	Chenildieu
48	Combeferre	Napoleon	Feuilly	Brevet	Combeferre	Brevet
49	Bahorel	Champmathieu	Combeferre	Cochepaille	Feuilly	Cochepaille
50	Joly	Chenildieu	Magnon	MmeHucheloup	Magnon	Isabeau
51	Prouvaire	Blacheville	Joly	Magnon	Joly	MmeDeR
52	BaronessT	MmeMagloire	Bahorel	MmeBurgon	Bahorel	Gervais
53	Grantaire	MotherInnocent	MmeBurgon	Prouvaire	MmeBurgon	Labarre
54	Magnon	Jondrette	Boulatruelle	BaronessT	Boulatruelle	Scaufflaire
55	MmeBurgon	OldMan	Child2	Grantaire	Child2	BaronessT
56	Child2	Prouvaire	Child1	Boulatruelle	Child1	MmePontmercy
57	Child1	Scaufflaire	Prouvaire	Child1	Prouvaire	Boulatruelle
58	Boulatruelle	MmeDeR	Perpetue	Child2	Perpetue	Child1
59	Perpetue	Dahlia	Grantaire	Perpetue	Grantaire	Child2
60	MmePontmercy	Judge	MmePontmercy	MmePontmercy	MmePontmercy	Perpetue
61	Fameuil	MmeHucheloup	Count	Favourite	Cravatte	Favourite
62	Listolier	Toussaint	Champtercier	Zephine	Napoleon	Zephine
63	Blacheville	Child2	Napoleon	Blacheville	OldMan	Dahlia
64	Favourite	MlleVaubois	Geborand	Dahlia	Champtercier	Blacheville
65	Zephine	CountessDeLo	CountessDeLo	Fameuil	Geborand	Listolier
66	Dahlia	Perpetue	OldMan	Listolier	CountessDeLo	Fameuil
67	MlleVaubois	Gribier	Cravatte	MlleVaubois	Count	MlleVaubois
68	Geborand	Listolier	MlleVaubois	Geborand	MlleVaubois	MotherPlutarch
69	Champtercier	Favourite	Listolier	Cravatte	Dahlia	Geborand
70	Cravatte	Marguerite	Blacheville	CountessDeLo	Fameuil	CountessDeLo
71	Napoleon	Boulatruelle	Zephine	Champtercier	Listolier	Cravatte
72	Count	Woman1	Dahlia	Count	Blacheville	Count
73	OldMan	BaronessT	Favourite	Napoleon	Favourite	Napoleon
74	CountessDeLo	Child1	Fameuil	OldMan	Zephine	Champtercier
75	MotherPlutarch	Woman2	Gribier	MotherPlutarch	Gribier	OldMan
76	Gribier	Cravatte	MotherPlutarch	Gribier	MotherPlutarch	Gribier
77	Jondrette	Gervais	Jondrette	Jondrette	Jondrette	Jondrette

Table 1: Ranking obtained with our method by varying the dissimilarity measure used in the coappearance network of characters in the novel Les Miserables.

Note that most measures lead us to similar rankings, however none are equal, as can be observed by a simple inspection of Table 1. From our point of view, the Meet/Min dissimilarity was that produced the worst results. We think the reason is that the normalization factor using the

corresponding dissimilarity measure is very permissive, considering two nodes fully similar when one of the inclusive neighbourhood is totally contained in the other. Furthermore, Meet/Min and Korbel dissimilarities does not to detect Javert as the second most important character in the network of Les Miserable, unlike the results obtained with the rest of disimilarity measures presented here.

3 Datasets

In the following we use as benchmark a set of databases of networks in which is known partially or totally the relevance of each node in the network. The centrality of each node is calculated and the most central nodes are showed in each case. We use the methodology presented in the paper, that is, we calculate the centrality of each node solving the eigenvector problem

$$c_i = \frac{1}{\lambda_{\max}} \sum_{j=1}^{n} W_{ij} c_j, \quad i = 1, 2, \cdots n \tag{13}$$

where $W_{ij} = A_{ij} \cdot D_{ij}$ and D_{ij} is the Jaccard dissimilarity matrix.

3.1 Florentine Marriages Network

This network was taken from [1], constructed through data from historical documents on the social relations among renaissance Florentine families. Bases on the analysis of the network, in [1, 2] a evidence is provided that support why the Medici were the most powerful family in the early of fifteenth century in Florence.

In Figures 1 and 2, we note that there is a difference between the ranking produced by contribution centrality and the other centrality measures, mainly emphasizing the difference between the results obtained with contribution centrality and those obtained with the eigenvector centrality, although both measures have similar heuristics. In Figure 3, the network is illustrated with the different centrality values produced by our method.

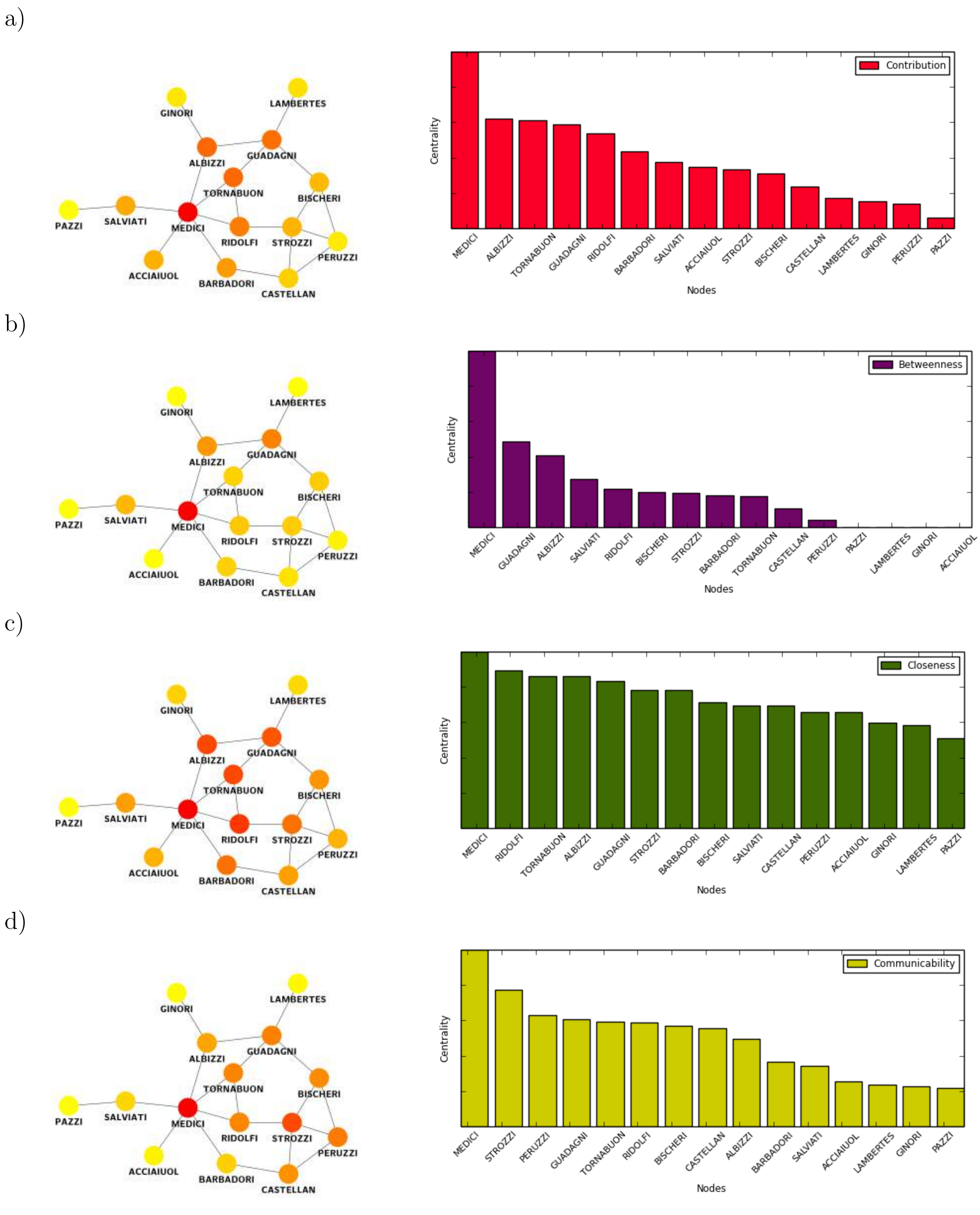

Figure 1: Centrality distributions of Florentine families network obtained by: a) Contribution, b) Betweenness, c) Closeness, d) Communicability

e)

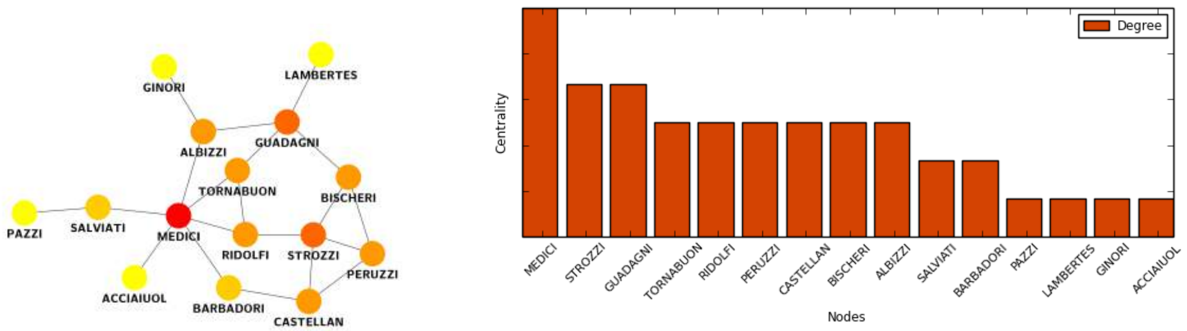

f)

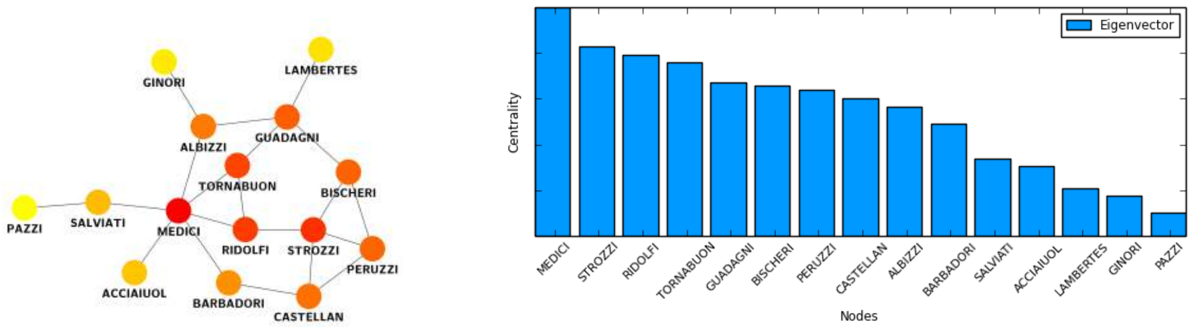

g)

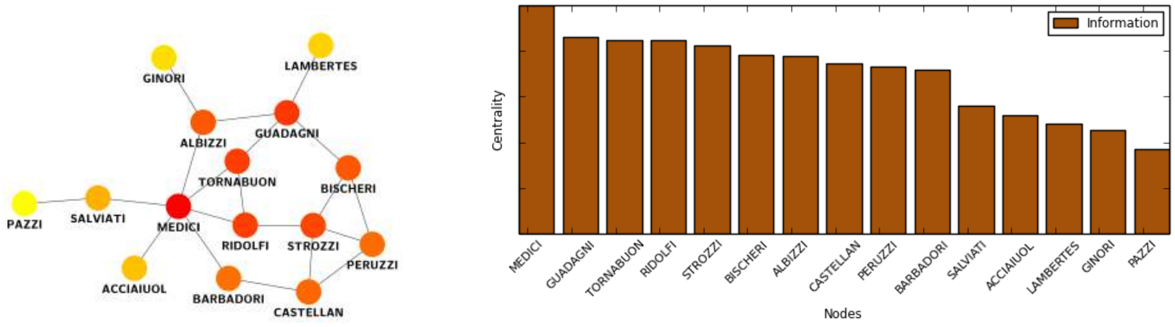

Figure 2: Centrality distributions of Florentine families network obtained by: e) Degree, f) Eigenvector, g) Information

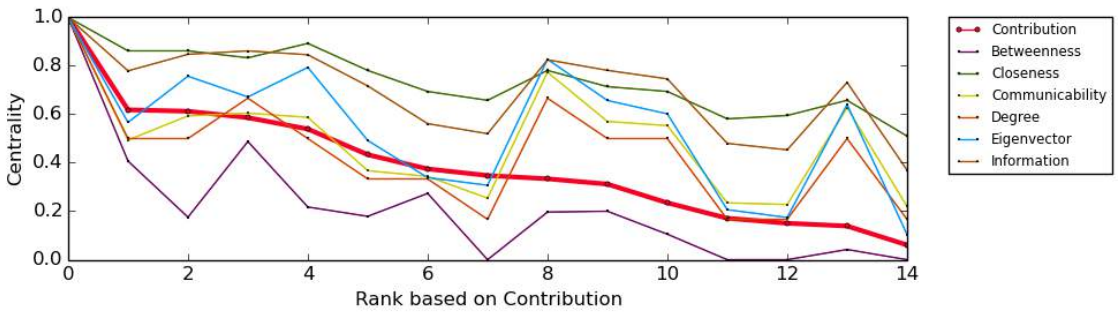

Figure 3: Behaviour of the different centrality measures in the network of Florentine families.

Table 2 shows nodes sorted by relevance according to different centrality measures.

Index	Contribution	Betweenness	Closeness	Communicability	Degree	Eigenvector	Information
1	Medici	Medici	Medici	Medici	Medici	Medici	Medici
2	Albizzi	Guadagni	Ridolfi	Strozzi	Strozzi	Strozzi	Guadagni
3	Tornabuon	Albizzi	Tornabuon	Peruzzi	Guadagni	Ridolfi	Tornabuon
4	Guadagni	Salviati	Albizzi	Guadagni	Tornabuon	Tornabuon	Ridolfi
5	Ridolfi	Ridolfi	Guadagni	Tornabuon	Ridolfi	Guadagni	Strozzi
6	Barbadori	Bischeri	Strozzi	Ridolfi	Peruzzi	Bischeri	Bischeri
7	Salviati	Strozzi	Barbadori	Bischeri	Castellan	Peruzzi	Albizzi
8	Acciaiuol	Barbadori	Bischeri	Castellan	Bischeri	Castellan	Castellan
9	Strozzi	Tornabuon	Salviati	Albizzi	Albizzi	Albizzi	Peruzzi
10	Bischeri	Castellan	Castellan	Barbadori	Salviati	Barbadori	Barbadori
11	Castellan	Peruzzi	Peruzzi	Salviati	Barbadori	Salviati	Salviati
12	Lambertes	Pazzi	Acciaiuol	Acciaiuol	Pazzi	Acciaiuol	Acciaiuol
13	Ginori	Lambertes	Ginori	Lambertes	Lambertes	Lambertes	Lambertes
14	Peruzzi	Ginori	Lambertes	Ginori	Ginori	Ginori	Ginori
15	Pazzi	Acciaiuol	Pazzi	Pazzi	Acciaiuol	Pazzi	Pazzi

Table 2: Comparative Ranking of nodes in the network of Florentine families with each centrality measure studied.

In this case, all measurements were able to detect the result obtained by [1, 2], i.e., the Medici family is the most important and influential in the network.

3.2 Zachary's Karate Club Network

This network was taken from [3] where the nodes were members of a university karate club and the links represented the presence of ties among the members of the club. For our proposes, we take only the topology, obviating the weight of the links.

In Figures 4 and 5, there is significant difference between the ranking produced by contribution centrality and the other centrality measures. Most methods detected the nodes 34, 33 and 1 as the main nodes, being this the correct ranking. Moreover, our method is the only one to point to node 33 above the node 1. We think that node 33 is in fact the second most important node in the network because certainly the student club is lead by the chief administrator given by the node 34 and the sub-officer given by the node 33, who decide to employed a part-time karate instructor, given by node 1, i.e., all nodes except 1 were students and members of the university club before its fission. Note that 33 and 34 belong to the larger community [16, 17], and node 33 strongly influences the node 34 due to its direct connection, and it shares much of the neighbourhood of this, i.e., 33 can assume the role of 34 in his absence. In this regard, we consider that 33 is the true top 2. Closeness, Eigenvector and Information placed node 3 above the node 33. The results obtained by contribution, betweenness, communicability and degree centralities indicate that node 3 we should be in position 4. In Figure 6, the network is illustrated with the different centrality values produced by contribution centrality and in Table 3 we show the rankings obtained through different centrality measures here compared.

a)

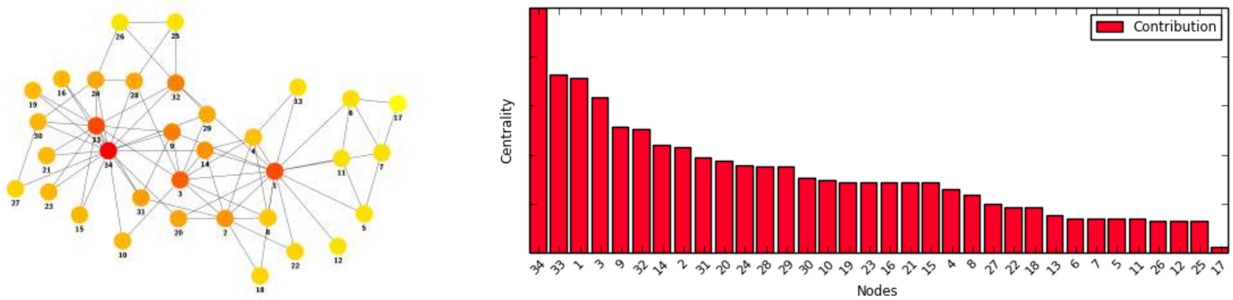

b)

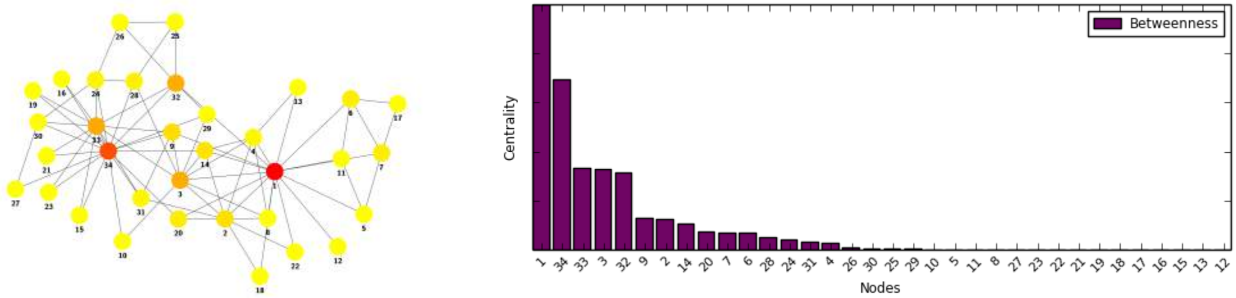

c)

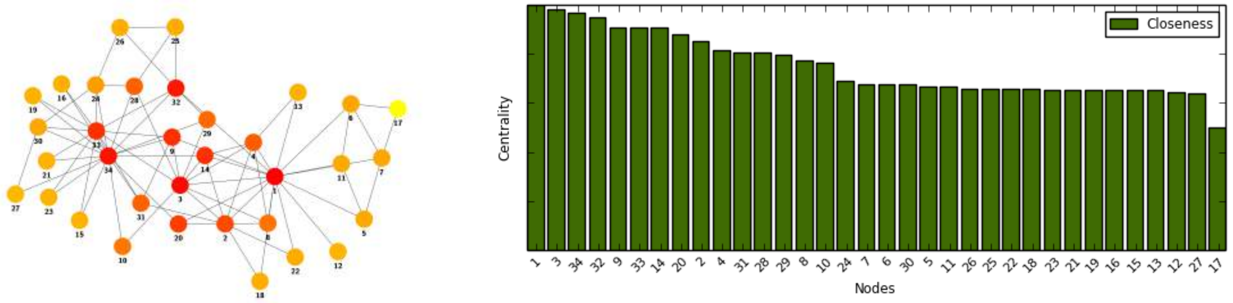

d)

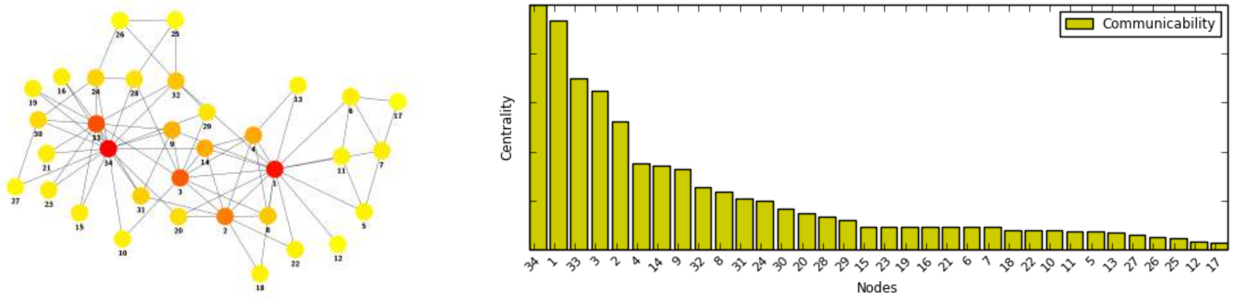

Figure 4: Centrality distributions of Zachary karate club network obtained by: a) Contribution, b) Betweenness, c) Closeness, d) Communicability

e)

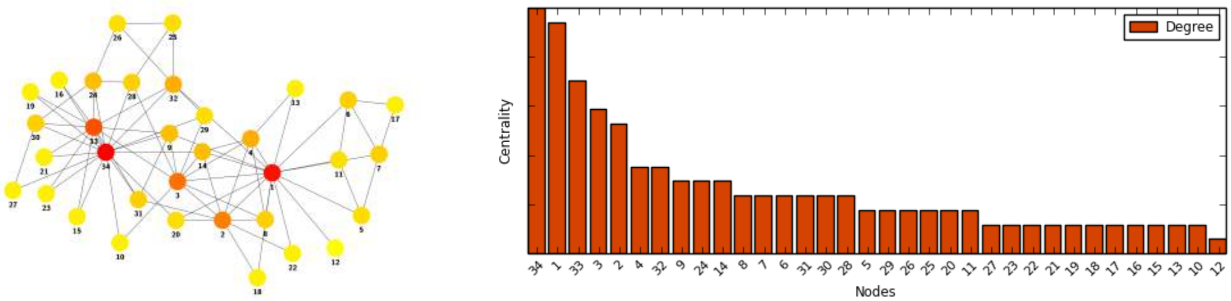

f)

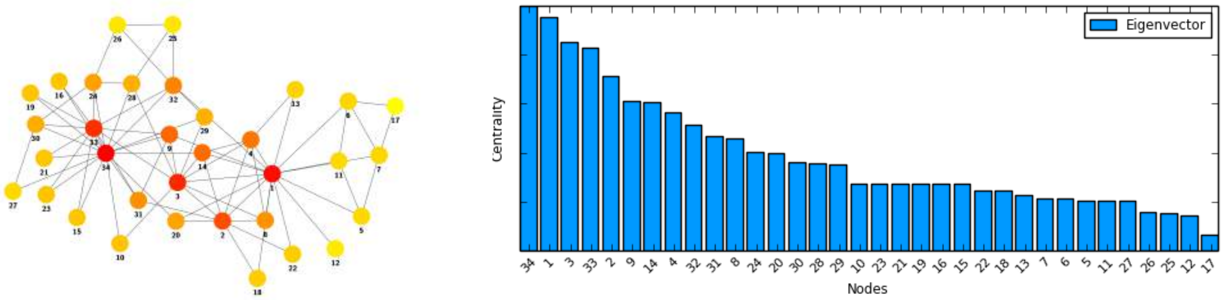

g)

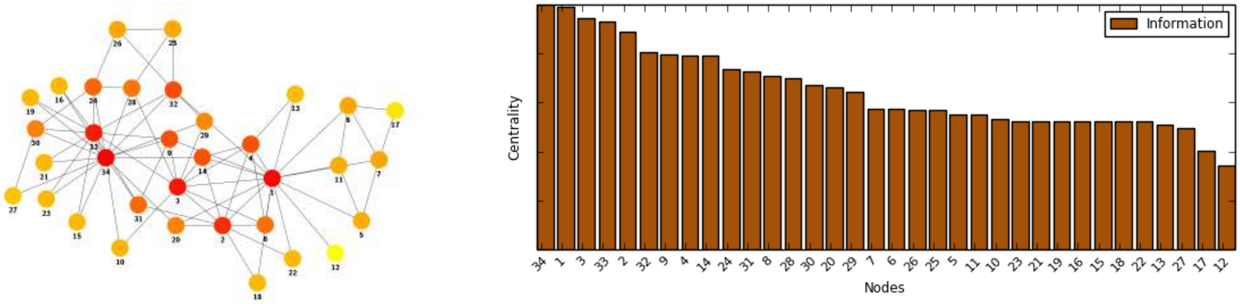

Figure 5: Centrality distributions of Zachary karate club network obtained by: e) Degree, f) Eigenvector, g) Information

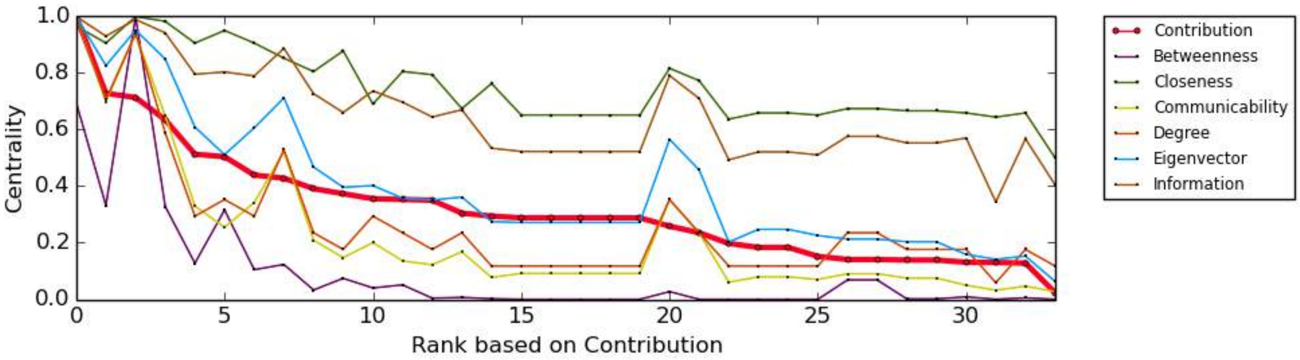

Figure 6: Behavior of the different centrality measures in the network of Zachary karate club .

Index	Contribution	Betweenness	Closeness	Communicability	Degree	Eienvector	Information
1	34	1	1	34	34	34	34
2	33	34	3	1	1	1	1
3	1	33	34	33	33	3	3
4	3	3	32	3	3	33	33
5	9	32	9	2	2	2	2
6	32	9	33	4	4	9	32
7	14	2	14	14	32	14	9
8	2	14	20	9	9	4	4
9	31	20	2	32	24	32	14
10	20	7	4	8	14	31	24

Table 3: Comparative Ranking of nodes in the network of Zachary karate club with each centrality measure studied.

3.3 Les Miserables Coappearances Network

We take the coappearances network of characters in Victor Hugo's novel "Les Miserables". Nodes represent characters, labeled by their names. Two characters are linked if these appear in the same chapter of the book [4]. We staying only with the topology, ignoring the number of such coappearances. It is well known that the most important characters are Valjean and Javert, having the roles of protagonist and antagonist, respectively. We can see the rankings obtained by each method in the Figures 7 and 8. It is very interesting note that eigenvector are unable to classify Valjean as the most important node, despite its initial heuristic remarkable likeness with contribution centrality. Communicability is another measure that are unable to detect Valjean as the most important. On the other hand, closeness and degree are unable to detect Javert as the second most important node. So, only contribution and information were able to correctly classify Valjean and Javert as the first and second most important characters in this novel. Finally, information centrality ranks Enjolras above Cossette, unlike our method, which is why we believe that contribution centrality generates a better ranking of the nodes in this network, illustrated in Figure 9. In Table 4 we show the rankings according to each centrality measure compared here.

a)

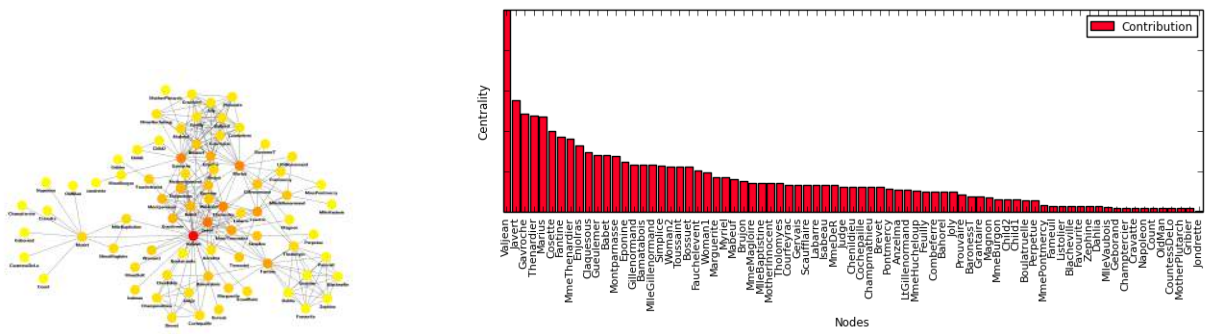

b)

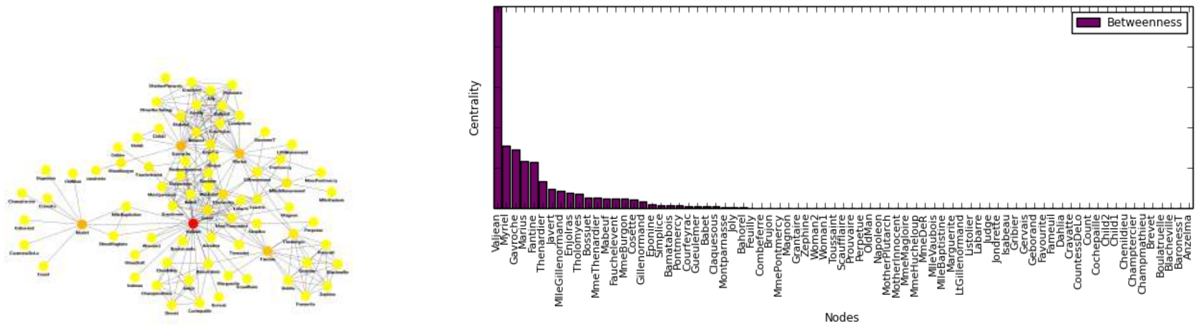

c)

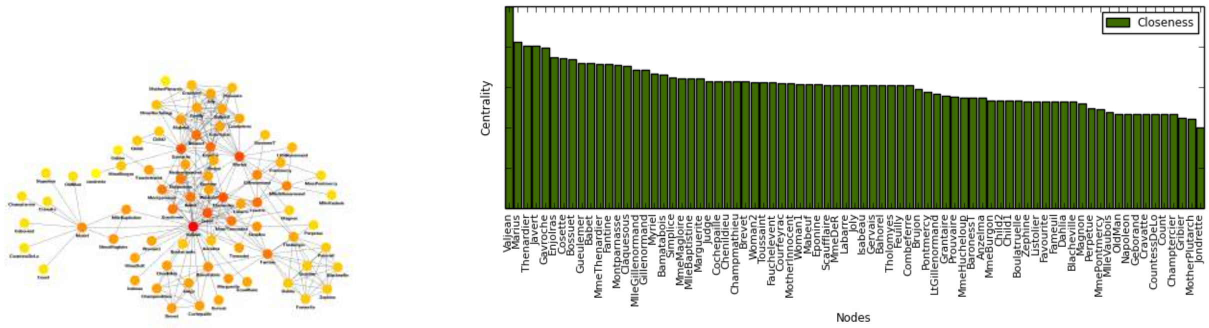

d)

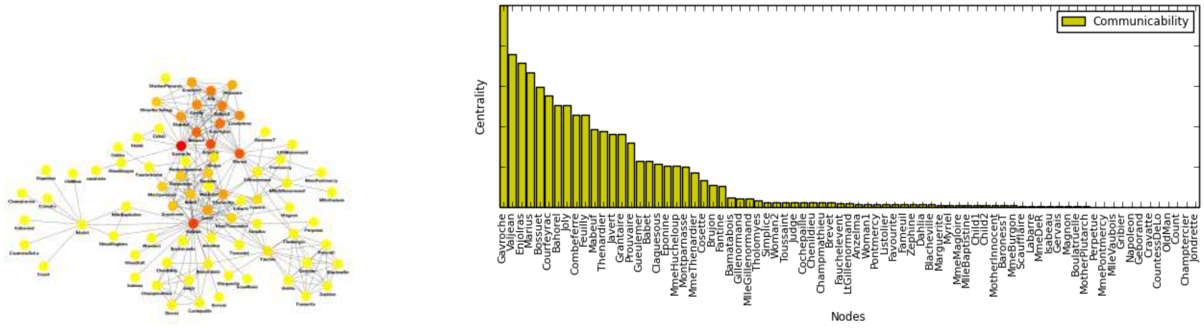

Figure 7: Centrality distributions of Les Miserables Coappearances network obtained by: a) Contribution, b) Betweenness, c) Closeness, d) Communicability.

e)

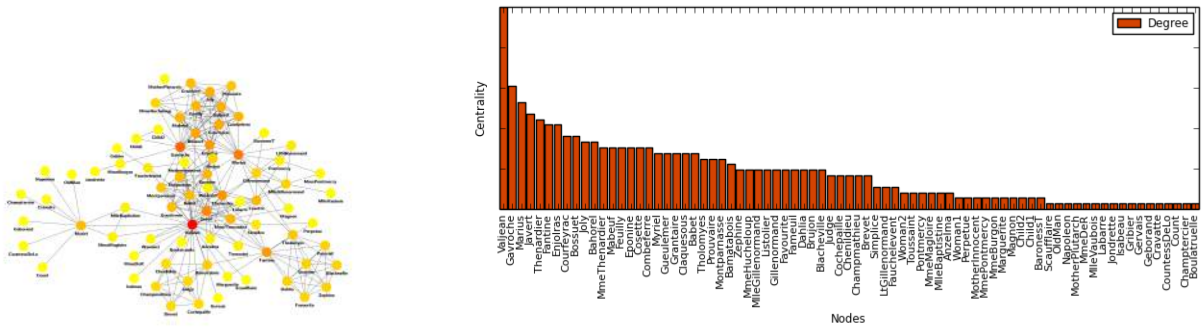

f)

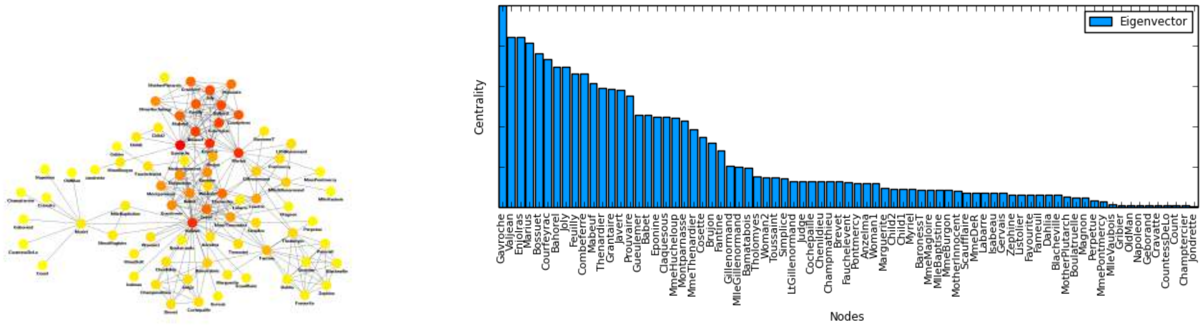

g)

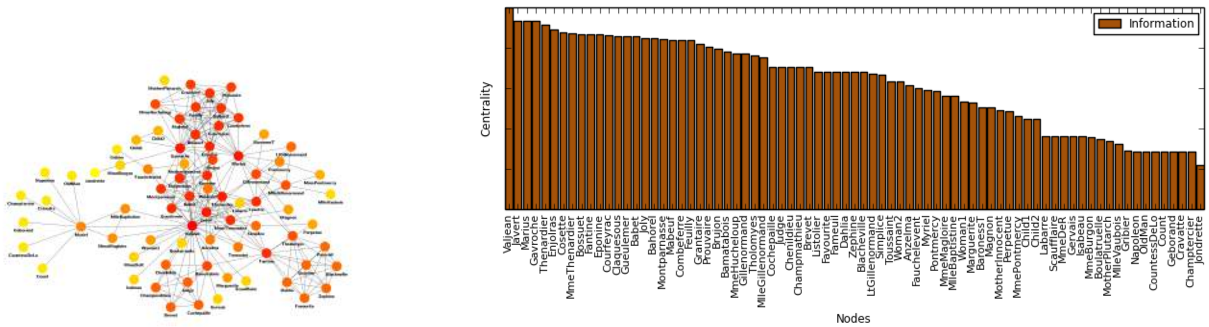

Figure 8: Centrality distributions of Les Miserables Coappearances network obtained by: e) Degree, f) Eigenvector, g) Information.

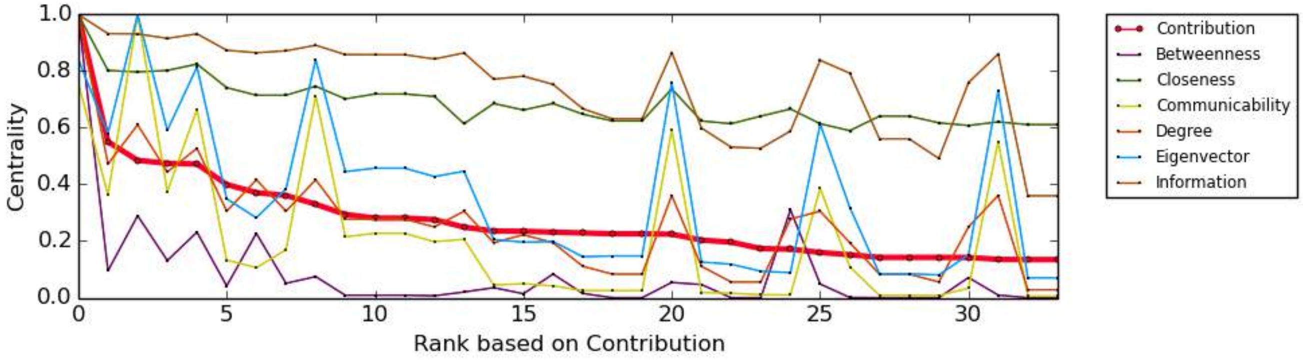

Figure 9: Behavior of the different centrality measures in the network of Les Miserables Coappearances.

Index	Contribution	Betweenness	Closeness	Communicability	Degree	Eienvector	Information
1	Valjean	Valjean	Valjean	Gavroche	Valjean	Gavroche	Valjean
2	Javert	Myriel	Marius	Valjean	Gavroche	Valjean	Javert
3	Gavroche	Gavroche	Thenardier	Enjolras	Marius	Enjolras	Marius
4	Thenardier	Marius	Javert	Marius	Javert	Marius	Gavroche
5	Marius	Fantine	Gavroche	Bossuet	Thenardier	Bossuet	Thenardier
6	Cosette	Thenardier	Enjolras	Courfeyrac	Fantine	Courfeyrac	Enjolras
7	Fantine	Javert	Cosette	Bahorel	Enjolras	Bahorel	Cosette
8	MmeThenardier	MlleGillenormand	Bossuet	Joly	Courfeyrac	Joly	MmeThenardier
9	Enjolras	Enjolras	Gueulemer	Combeferre	Bossuet	Feuilly	Bossuet
10	Claquesous	Tholomyes	Babet	Feuilly	Joly	Combeferre	Fantine

Table 4: Comparative Ranking of nodes in the network of Les Miserables Coappearances with each centrality measure studied.

3.4 Dolphin social network

This is a social network of bottlenose dolphins where the nodes are the bottlenose dolphins (genus Tursiops) of a community living off Doubtful Sound and a edge between dolphins indicates a frequent association. The dolphins were observed between 1994 and 2001 and was reported in [5]. In this case, it is known that the three most important dolphins are SN4, Grin and Topless. However, betweenness coefficient tells us that Grin and topless have less intermediation than SN4 in the network, appearing for this measure in the 4 rank (see Table 5). Also, for closeness SN4 is more closer to the rest of the other nodes in the network in comparison with Topless and Grin, as shown in Table 5. Moreover, a recent work suggest that SN4 is the most central node in the network, by using the Path Score (PS) [18]. Thus, we see that with the inclusion of dissimilarity measure is achieved detect more subtle details than those achieved with the classical measures. If we sum the rankings for each measure and average them we obtain again that SN4 is the top 1 and if we study the community structure of this network, we see that SN4 is in the bigger community [17]. In Figures 10 and 11, we can see the rankings produced by each centrality measure here explored. In Figure 12, we represent the centrality of each node produced by our method in the network.

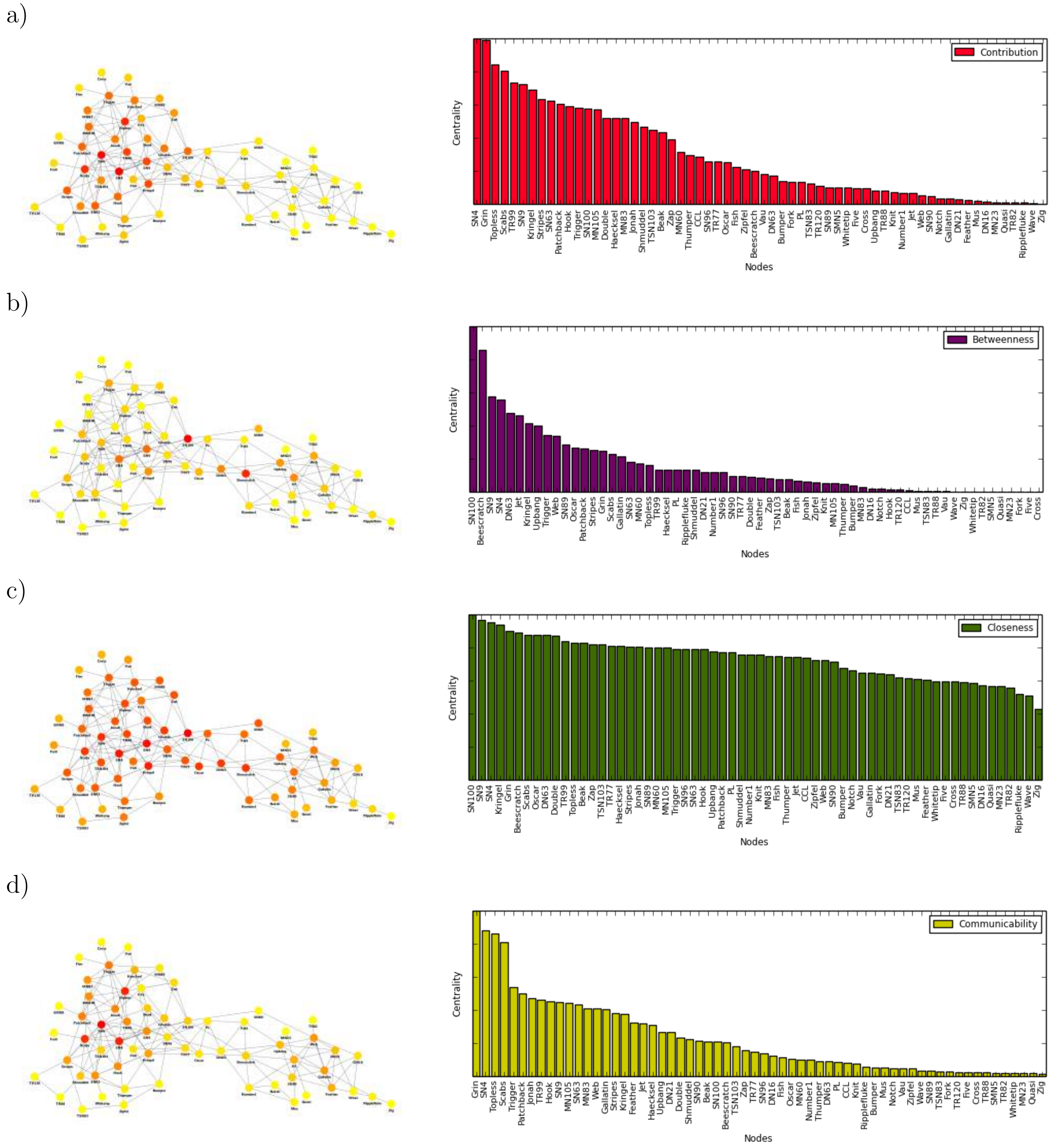

Figure 10: Centrality distributions of Dolphin social network obtained by: a) Contribution, b) Betweenness, c) Closeness, d) Communicability.

e)

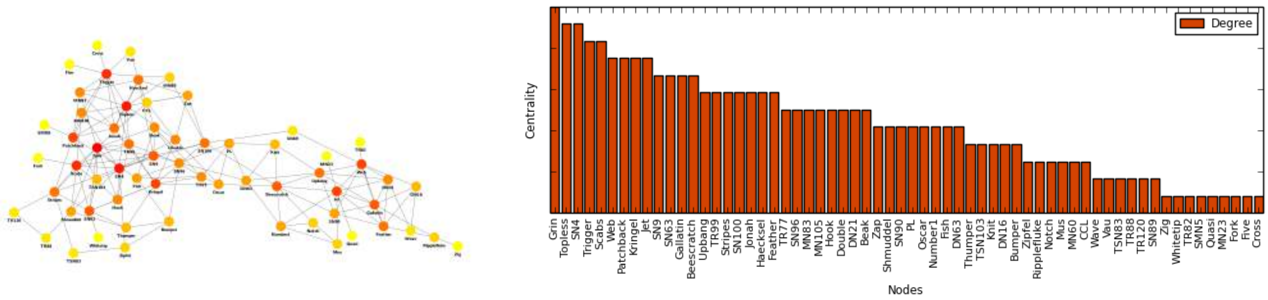

f)

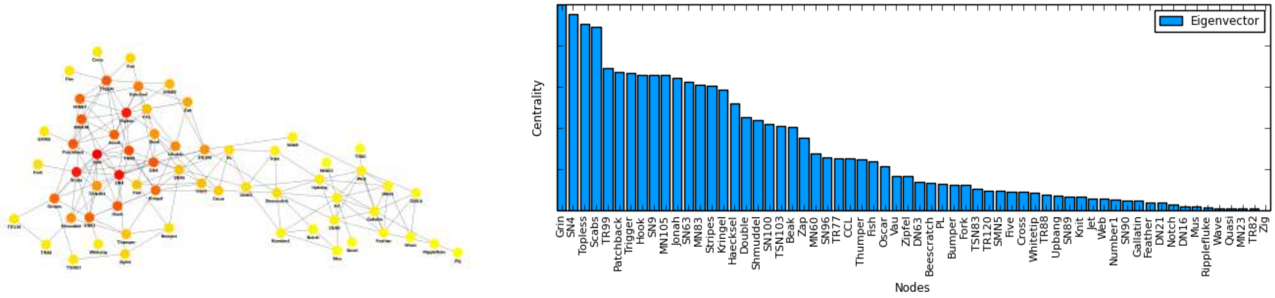

g)

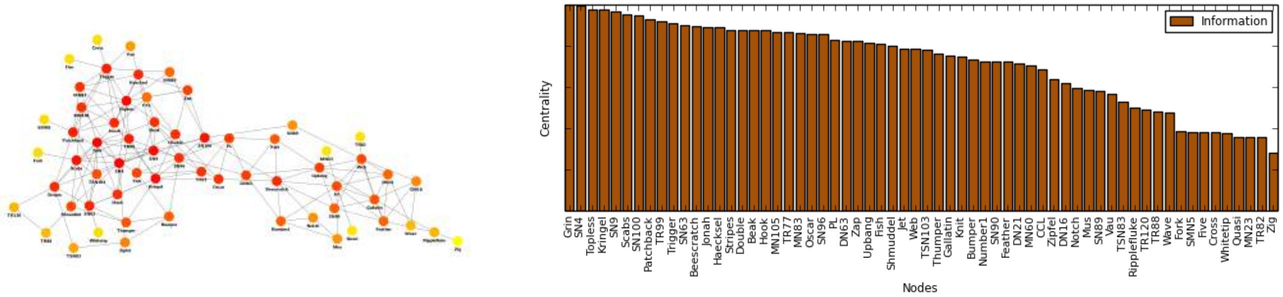

Figure 11: Centrality distributions of Dolphin social network, obtained by: e) Degree, f) Eigenvector, g) Information.

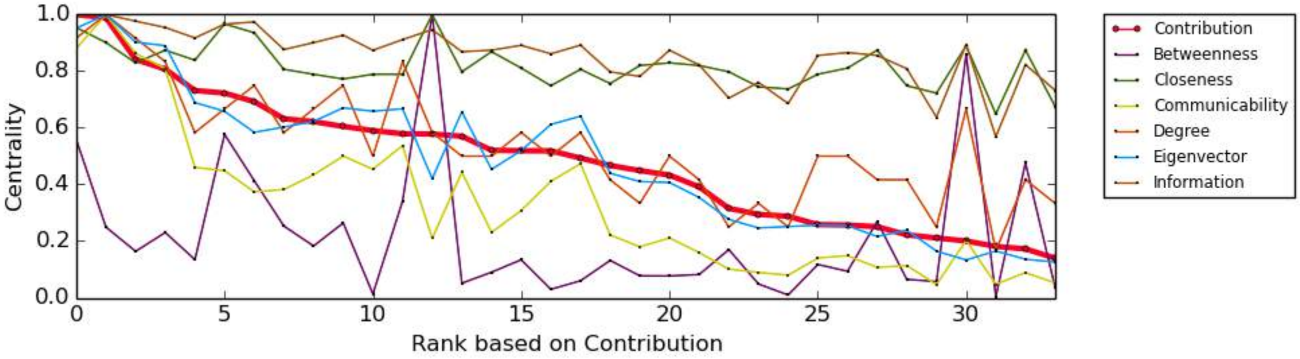

Figure 12: Behavior of the different centrality measures in the network of Dolphin social.

Index	Contribution	Betweenness	Closeness	Communicability	Degree	Eienvector	Information
1	SN4	SN100	SN100	Grin	Grin	Grin	Grin
2	Grin	Beescratch	SN9	SN4	Topless	SN4	SN4
3	Topless	SN9	SN4	Topless	SN4	Topless	Topless
4	Scabs	SN4	Kringel	Scabs	Trigger	Scabs	Kringel
5	TR99	DN63	Grin	Trigger	Scabs	TR99	SN9
6	SN9	Jet	Beescratch	Patchback	Web	Patchback	Scabs
7	Kringel	Kringel	Scabs	Jonah	Patchback	Trigger	SN100
8	Stripes	Upbang	Oscar	TR99	Kringel	Hook	Patchback
9	SN63	Trigger	DN63	Hook	Jet	SN9	TR99
10	Patchback	Web	Double	SN9	SN9	MN105	Trigger

Table 5: Comparative Ranking of nodes in the network of Dolphin social with each centrality measure studied.

3.5 Terrorist Network

This network shows the contacts between suspected terrorists involved in the bombing of train in Madrid on March 11, 2004 [19]. A node represents a terrorist and two terrorist are linked if there was a contact between them. The main material author was Jamal Zougam [20], accused and was sentenced to 34,715 years [21]. The second in the ranking is Mohamed Chaoui, half-brother of Jamal Zougam [22], accused of purchase the thirteen mobile phone SIM cards [23] used to detonate the explosive device. In third position appears Imad Eddin Barakat (also known as Abu Dahdah), sentenced to twenty-seven years in prison for his participation in the September 11th terror attacks. Spanish intelligence officer Rafael Gomez Menor speculated that Imad Eddin Barakat oversaw the planning of the train bombings, as intellectual author [24]. Thus, almost all centrality measures seems to have correctly identified the main members related with the planning and execution of this terrorist attack, with the exception of closeness and betweenness that ranked Imad Eddin Barakat in the positions fifth and fourth respectively, as we can see in Tables 6 and 7. In Figures 13 and 14, we can see more detailed differences between the rankings. In Figure 15 is illustrated the centrality of each node through contribution centrality.

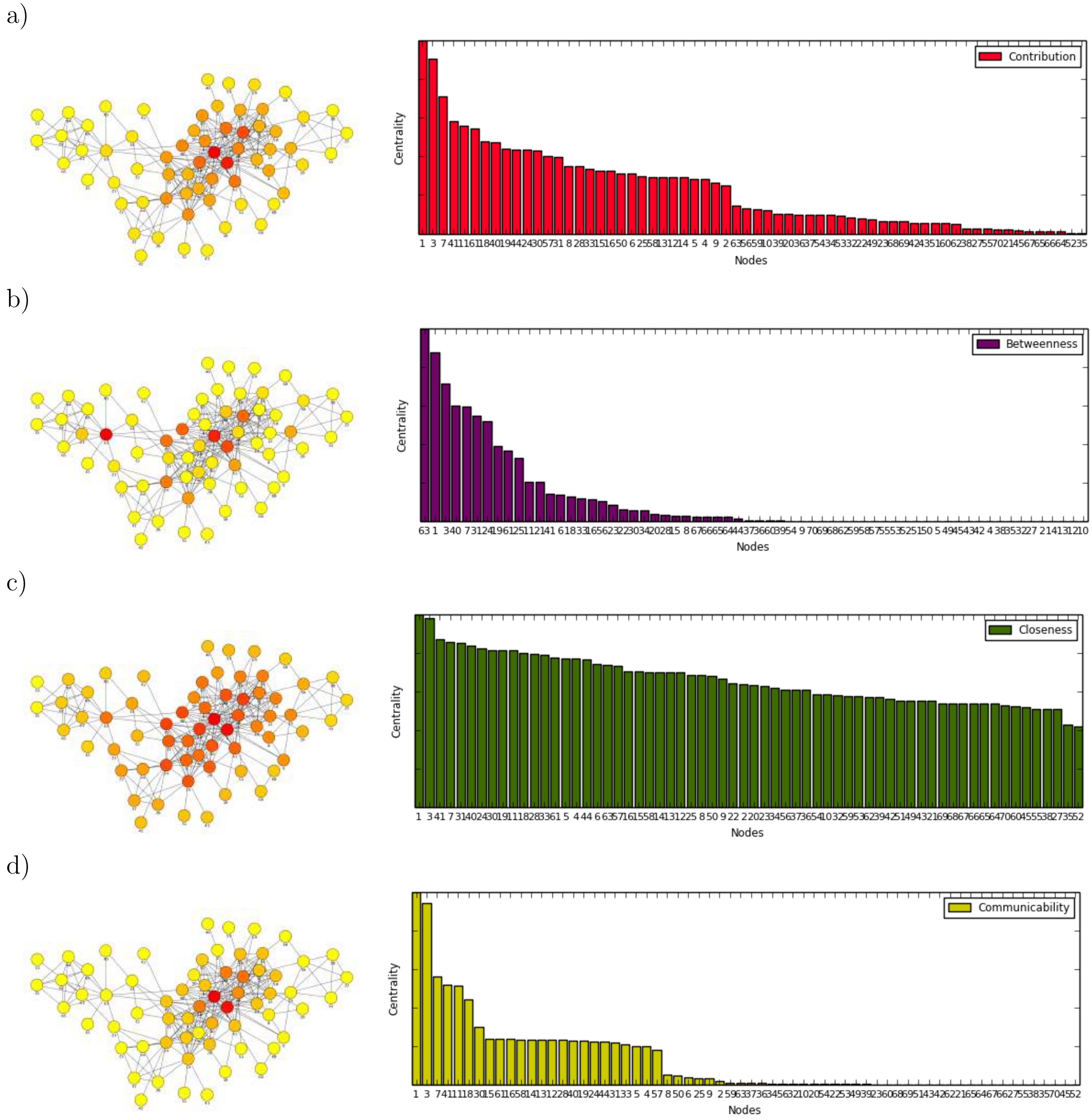

Figure 13: Centrality distributions of communication network among those involved in the terrorist attack in Madrid subway (M12) obtained by: a) Contribution, b) Betweenness, c) Closeness, d) Communicability.

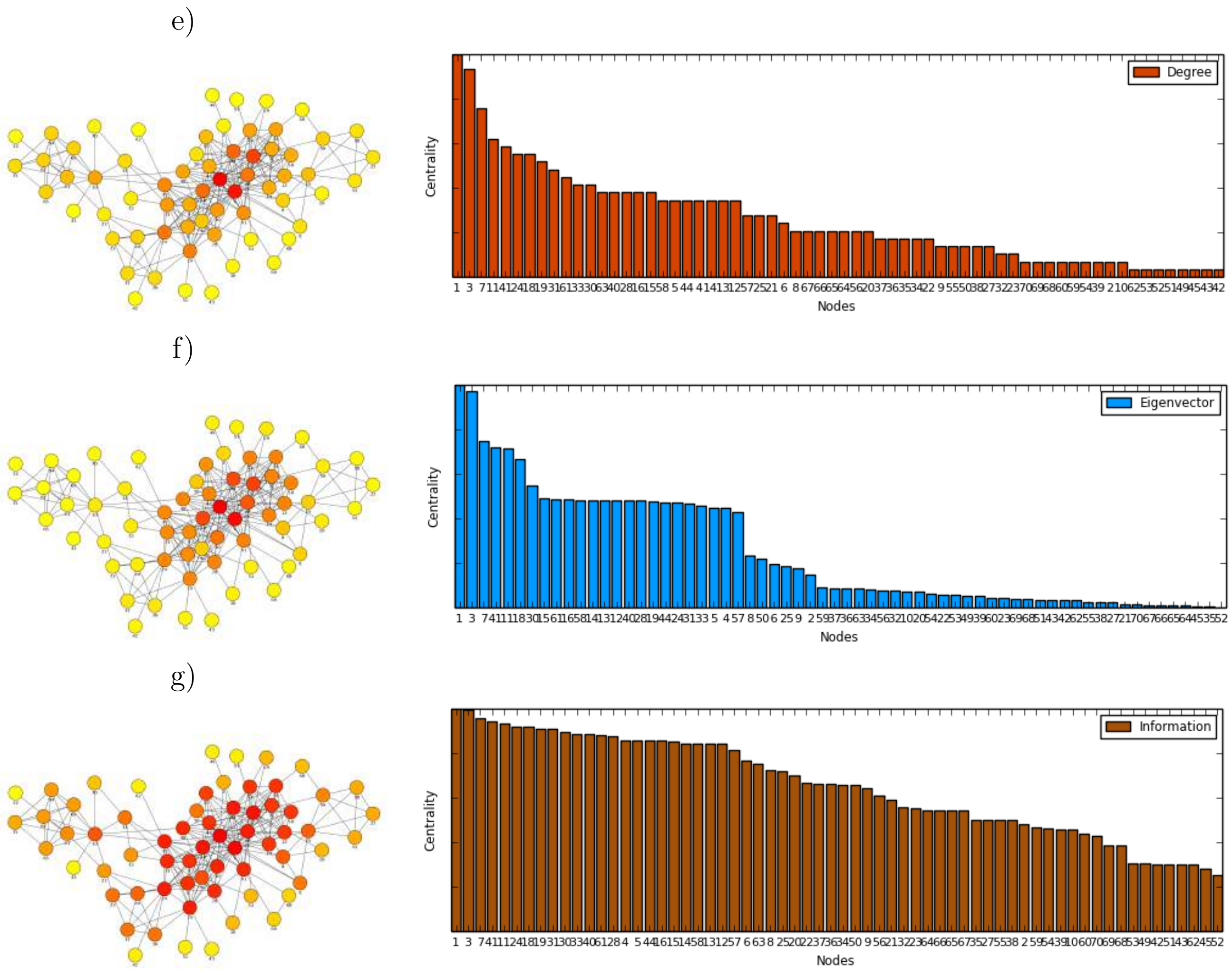

Figure 14: Centrality distributions of communication network among those involved in the terrorist attack in Madrid subway (M12) obtained by: e) Degree, f) Eigenvector, g) Information.

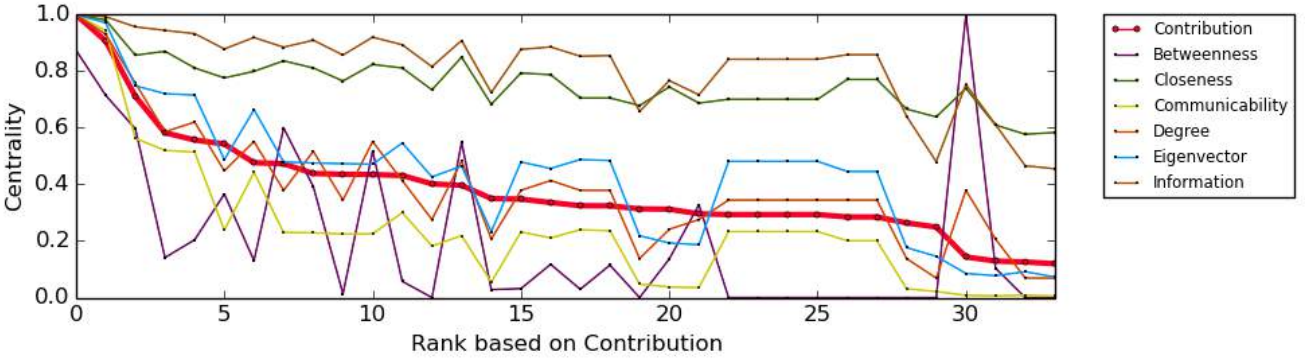

Figure 15: Behavior of the different centrality measures in the network of the terrorist attack in Madrid subway (M12).

Index	Contribution	Betweenness	Closeness	Communicability	Degree	Eienvector	Information
1	1	63	1	1	1	1	1
2	3	1	3	3	3	3	3
3	7	3	41	7	7	7	7
4	41	40	7	41	11	41	41
5	11	7	31	11	41	11	11
6	61	31	40	18	24	18	24
7	18	24	24	30	18	30	18
8	40	19	30	15	19	15	19
9	19	61	19	61	31	61	31
10	44	25	11	16	61	16	30
11	24	11	18	58	33	58	33
12	30	21	28	14	30	14	40
13	57	41	33	13	63	13	61
14	31	6	61	12	40	12	28
15	8	18	5	28	28	40	4
16	28	33	4	40	16	28	5
17	33	16	44	19	15	19	44
18	15	56	6	24	58	44	16
19	16	23	63	44	5	24	15
20	50	22	57	31	44	31	14

Table 6: Top 20 most central terrorist for each centrality measure.

1	Jamal Zougam	2	Mohamed Bekkali	3	Mohamed Chaoui
4	Vinay Kholy	5	Suresh Kumar	6	Mohamed Chedadi
7	Imad Eddin Barakat	8	Abdelaziz Benyaich	9	Abu Abderrahame
10	Omar Dhegayes	11	Amer Azizi	12	Abu Musad Alsakaoui
13	Mohamed Atta	14	Ramzi Binalshibh	15	Mohamed Belfatmi
16	Said Bahaji	17	Alí Amrous	18	Galeb Kalaje
19	Abderrahim Zbakh	20	Farid Oulad Ali	21	José Emilio Suárez
22	Khalid Ouled Akcha	23	Rafa Zuher	24	Naima Oulad Akcha
25	Abdelkarim el Mejjati	26	Abdelhalak Bentasser	27	Anwar Adnan Ahmad
28	Basel Ghayoun	29	Faisal Alluch	30	S B Abdelmajid Fakhet
31	Jamal Ahmidan	32	Said Ahmidan	33	Hamid Ahmidan
34	Mustafa Ahmidan	35	Antonio Toro	36	Mohamed Oulad Akcha
37	Rachid Oulad Akcha	38	Mamoun Darkazanli	39	Fouad El Morabit Anghar
40	Abdeluahid Berrak	41	Said Berrak	42	Waanid Altaraki Almasri
43	Abddenabi Koujma	44	Otman El Gnaut	45	Abdelilah el Fouad
46	Mohamad Bard Ddin Akkab	47	Abu Zubaidah	48	Sanel Sjekirika
49	Parlindumgan Siregar	50	El Hemir	51	Anuar Asri Rifaat
52	Rachid Adli	53	Ghasoub Al Albrash	54	Said Chedadi
55	Mohamed Bahaiah	56	Taysir Alouny	57	OM. Othman Abu Qutada
58	Shakur	59	Driss Chebli	60	Abdul Fatal
61	Mohamed El Egipcio	62	Nasredine Boushoa	63	Semaan Gaby Eid
64	Emilio Llamo	65	Ivan Granados	66	Raul Gonzales Perez
67	El Gitanillo	68	Moutaz Almallah	69	Mohamed Almallah
70	Yousef Hichman				

Table 7: Dictionary of labels for terrorist network.

3.6 Airport USA 97

This is a model of commercial air traffic network among airports in the United States [6]. The network consist of 332 nodes (representing airports) and 2126 links, where each link is associated connecting airports through a direct flight. It is important to study the centrality of this network, since the hubs are related to points of optimal spread of disease, which is a serious public health problem. Detect such nodes would prevent or control the spread of diseases at local and global scale. The hubs are also related to vulnerability points in the airport network (points that in case of failure hinder all flights) what is of interest for airlines, since the flight delay produce losses. Detecting and preventing problems involve efficient and safe transportation. Only using the underlying topology and without information about number of passengers, charges transported or any other statistics/weights, we observe that contribution centrality detects within top 4 Chicago O'hare Intl., Dallas/Fort Worth Intl. and The William B. Hartsfield Atlanta that are three of the four major airports to date [25], missing only the Los Angeles Intl. In this case, only betweenness centrality obtain two of the four most important airports, meanwhile others have a similar ranking, as we can see in Table 8 where the nodes appears encoded by numbers and the translation of the names in Table 9. Finally, in Figures 16, we can see the profile of the centrality values for each measure, showing in Figure 17 the network with the different centrality values produced by contribution centrality.

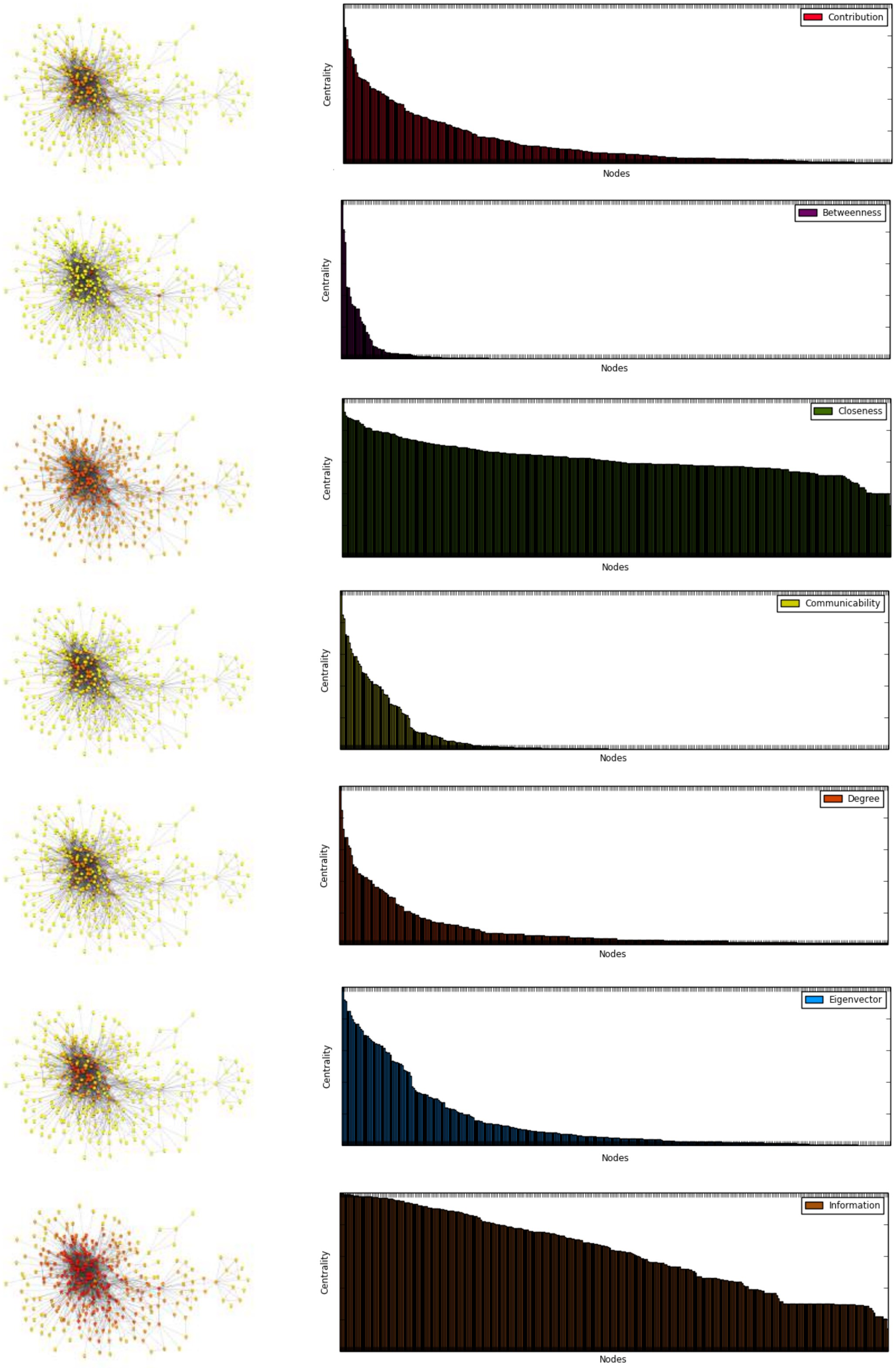

Figure 16: Centrality distributions of air traffic network, obtained by the various methods considered.

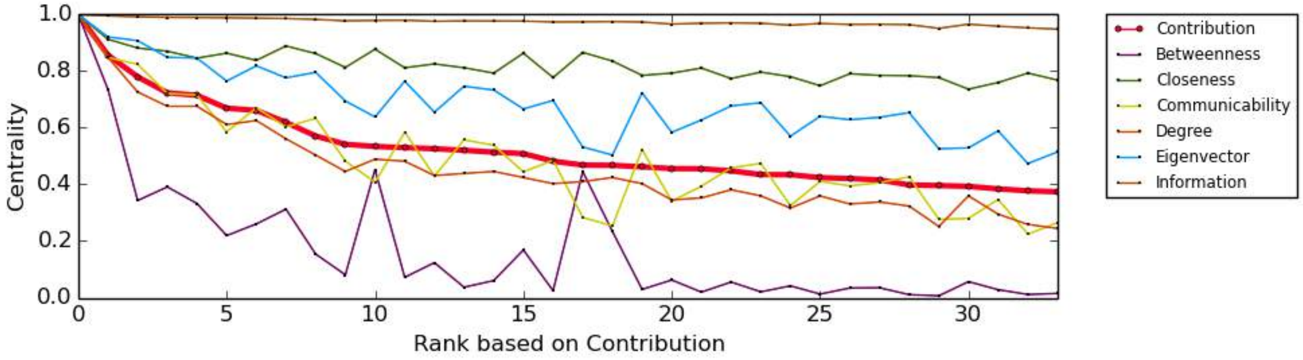

Figure 17: Behavior of the different centrality measures.

Index	Contribution	Betweenness	Closeness	Communicability	Degree	Eienvector	Information
1	118	118	118	118	118	118	118
2	261	8	261	261	261	261	261
3	255	261	67	255	255	255	255
4	182	201	255	182	182	182	182
5	152	47	201	152	152	152	152
6	166	182	182	230	230	230	166
7	230	255	47	112	166	112	230
8	67	152	248	67	67	67	67
9	112	313	166	166	112	166	112
10	293	13	112	147	201	147	147
11	201	67	152	176	147	176	201
12	147	230	230	162	293	162	293
13	258	144	144	299	162	299	176
14	176	166	258	217	176	217	162
15	162	65	293	293	258	293	248
16	248	248	176	109	248	109	258
17	217	112	147	174	144	174	144
18	47	258	219	248	47	248	47
19	144	329	109	258	299	258	217
20	299	293	263	131	217	131	299

Table 8: Comparative Ranking of nodes in the network of air traffic with each centrality measure studied.

8	Anchorage Intl	13	Bethel
47	Seattle-Tacoma Intl	65	Portland Intl
67	Minneapolis-St Paul Intl/Wold	109	General Edward Lawrence Logan
112	Detroit Metropolitan Wayne Cou	118	Chicago O'hare Intl
131	Cleveland-Hopkins Intl	144	Salt Lake City Intl
147	Newark Intl	152	Pittsburgh Intll
162	Philadelphia Intl	166	Stapleton Intl
174	Baltimore-Washington Intl	176	Cincinnati/Northern Kentucky I
182	Lambert-St Louis Intl	201	San Francisco Intl
217	Nashville Intll	219	Mc Carran Intl
230	Charlotte/Douglas Intl	248	Los Angeles Intl
255	The William B Hartsfield Atlan	258	Phoenix Sky Harbor Intl
261	Dallas/Fort Worth Intl	263	San Diego Intl-Lindbergh Fld
293	Houston Intercontinental	299	Orlando Intl
313	Honolulu Intl	329	Guam Intll

Table 9: Dictionary of labels for airports network.

4 Runtime performance

An analysis of the performance of our method was performed in a set of Barabási - Albert networks [31], with a number of nodes distributed logarithmically from 10^2 to 10^5 and parameter $k = 2$. For each N, were taken 10 networks and the runtime of our algorithm was averaged. The results obtained are shown in Table 10 and in Figure 18.

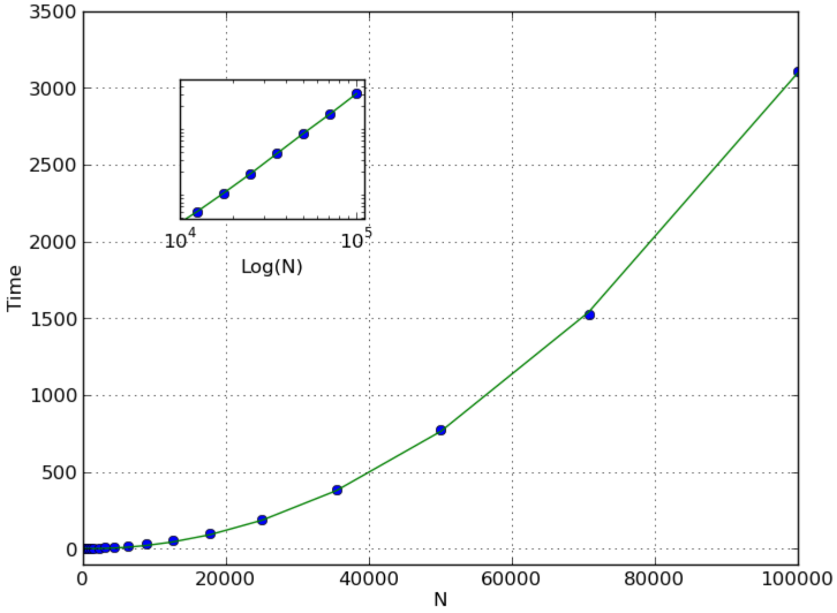

Figure 18: Average runtimes of our method implemented in Python. The inset is in log-log scale.

Runtimes were quite short, with 50 minutes for the largest benchmark network. The software used for such simulations was written in Python, which is well known to have a performance 20 times lower for intensive calculations, compared to languages like C/C++, or Fortran, so we

**Size**	**Time (Sec.)**
100	0.16
141	0.18
199	0.25
281	0.35
398	0.50
562	0.72
794	1.06
1122	1.57
1584	2.38
2238	3.68
3162	5.82
4466	9.53
6309	15.98
8912	27.69
12589	50.46
17782	96.46
25118	188.22
35481	382.17
50118	774.35
70794	1523.48
100000	3106.19

Table 10: Sizes and average runtimes of each network taken from the Barabási-Albert model.

estimate that analyse a network of 10^5 nodes with a code written and optimized in C/C++ or Fortran will not exceed 3 minutes, allowing process networks in the range of million nodes in a few minutes. After analysing all these networks, it was possible to detect hub as the most important node in each network as we expect.

References

[1] Padgett, J.F. & Ansell, C.K. Robust Action and the Rise of the Medici, 1400-1434. *American Journal of Sociology.* **98**, 1259 (1993).

[2] Matthew J. *Social and Economic Networks.* (Princeton University Press, 2010).

[3] Zachary W. An information flow model for conflict and fission in small groups. *Journal of Anthropological Research*, **33**, 452-473, (1977).

[4] Knuth D. E. *The Stanford GraphBase: A Platform for Combinatorial Computing.* (Addison-Wesley, Reading, MA 1993).

[5] Lusseau, D. et al. The bottlenose dolphin community of doubtful sound features a large proportion of long-lasting associations: Can geographic isolation explain this unique trait?. *Behavioral Ecology and Sociobiology.* **54**, 396-405 (2003).

[6] Batageli V. & Mrvar A. *Pajek Datasets* available at `http://vlado.fmf.uni-lj.si/pub/networks/data/default.htm`

[7] Levandowsky M. & Winter D. Distance between sets. *Nature.* **234** (5), 34–35 (1971).

[8] Goldberg, D.S. & Roth, F.P. Assessing experimentally derived interactions in a small world. *Proceedings of the National Academy of Sciences*, **100**, 4372-4376 (2003).

[9] Ravasz, E., Somera, A.L., Mongru, D.A., Oltvai, Z.N. & Barabási, A.L. Hierarchical organization of modularity in metabolic networks. *Science*, **297**, 1551-1555 (2002).

[10] Sørensen, T. A method of establishing groups of equal amplitude in plant sociology based on similarity of species and its application to analyses of the vegetation on Danish commons. *Kongelige Danske Videnskabernes Selskab*, **5** (4), 1–34 (1957).

[11] Mirkin, B. & Koonin, E. A top-down method for building genome classification trees with linear binary hierarchies. Bioconsensus: DIMACS Working Group Meetings on Bioconsensus: October 25-26, 2000 and October 2-5, 2001. *DIMACS Center*, **61**, 97 (2003).

[12] Brun, C., Herrmann, C. & Guénoche, A. Clustering proteins from interaction networks for the prediction of cellular functions. *BMC bioinformatics*, **5**, 95 (2004).

[13] Korbel, J.O., Snel, B., Huynen, M.A. & Bork, P. SHOT: A web server for the construction of genome phylogenies. *Trends in Genetics*, **18**, 158-162 (2002).

[14] Dalgard P. *Introductory Statistics with R, Statistics and computing Series.* (Springer, 2008).

[15] Cover, T.M. and Thomas, J.A. *Elements of information theory.* (John Wiley & Sons, (1991)).

[16] Newman, M. Modularity and community structure in networks. *Proceedings of the National Academy of Sciences*, **103**, 8577-8582 (2006).

[17] Fortunato, S. Community detection in graphs. *Physics Reports*, **486**, 75-174 (2010).

[18] Lee, S.H., Cucuringu, M. & Porter, M. a Density-based and transport-based core-periphery structures in networks. *Physical Review E*, **89**, 032810 (2014).

[19] Hayes, B. Connecting the dots: can the tools of graph theory and social-network studies unravel the next big plot?. *American Scientist*, **94** (5), 400-404 (2006).

[20] BBC, "Jamal Zougam: Madrid bomb suspect". 18 March 2004. Available at http://news.bbc.co.uk/2/hi/europe/3515790.stm

[21] Judgment 503/2008 of the Criminal Chamber of the Supreme Court avaliable at http://www.elpais.com/elpaismedia/ultimahora/media/200807/17/espana/20080717elpepunac_1_Pes_PDF.doc

[22] Gaynor T., Madrid Bomb Suspects are Linked to Attacks on Twin Towers, *The Independent*, (2004). Available at http://www.independent.co.uk/news/world/europe/madrid-bomb-suspects-are-linked-to-attacks-on-twin-towers-566359.html

[23] BBC News Correspondent, Madrid Bombings: The Investigation, *BBC News*, (2004). Available at http://news.bbc.co.uk/1/shared/spl/hi/europe/05/madrid_bombings/html/2.stm

[24] Rodríguez J. A., El comisario de terrorismo islamista considera a Abu Dahdah el autor intelectual del 11-M, *El Pais*, (2004). Available at http://elpais.com/diario/2004/10/26/espana/1098741608_850215.html

[25] Federal Aviation Administration, List of Commercial Service Airports based on CY2013 Enplanements, June 20, 2014. Available at http://www.faa.gov/airports/planning_capacity/passenger_allcargo_stats/passenger/media/preliminary-cy13-commercial-service-enplanements.pdf

[26] Newman, M.E.J. A measure of betweenness centrality based on random walks. *Social Networks* **27**, 39-54 (2005).

[27] Lee, S., Yook, S.H. & Kim, Y. Centrality measure of complex networks using biased random walks. *European Physical Journal B* **68**, 277-281 (2009).

[28] Rosvall, M. & Bergstrom, C.T. Maps of random walks on complex networks reveal community structure. *Proceedings of the National Academy of Sciences* **105**, 1118-1123 (2008).

[29] Pons, P. & Latapy, M. Computing Communities in Large Networks Using Random Walks. Journal of Graph Algorithms and Applications 10, 191-218 (2006).

[30] http://benchmarksgame.alioth.debian.org/

[31] Barabási, A. & Réka, A. Emergence of scaling in random networks. *Science* **286** (5439), 509–512 (1999).
